# Use of molecular networking to identify 2,5-diketopiperazines in chocolates as potential markers of bean variety

**DOI:** 10.1016/j.heliyon.2022.e10770

**Published:** 2022-09-27

**Authors:** Amandine André, Bettina Casty, Lisa Ullrich, Irene Chetschik

**Affiliations:** ZHAW Zurich University of Applied Sciences, School of Life Sciences and Facility Management, ILGI Institute of Food and Beverage Innovation, Research Group Food Chemistry, 8820, Wädenswil, Switzerland

**Keywords:** 2,5-Diketopiperazines, Cacao, Chocolate, Cocoa, Criollo, Forastero, HPLC-MS/MS, Molecular networks, Trinitario

## Abstract

2,5-diketopiperazines are cyclic dipeptides found, among others, in chocolate. Although those compounds are contributing greatly to its pleasant bitterness, they can also be seen as interesting markers of cocoa beans processing. To evaluate the influence of bean variety and processing technology on the quantity of 2,5-diketopiperazines formed in chocolates, HPLC-MS/MS analyses were conducted, and a molecular network was built with the MS^2^ data. This approach eases the identification of 2,5-diketopiperazines within complex datasets and allows to visualize the chemical diversity of all samples. Using this methodology, 33 dark chocolates were analysed. 18 different diketopiperazine were identified and quantified. Among them, cyclo(L-ile-L-val), cyclo(L-leu-L-ile) and cyclo(L-phe-L-phe) were, to the best of our knowledge, detected for the first time in chocolate. The molecular network allows the clear visualization of differences between samples. The principal component analysis revealed the clustering of small batch chocolate samples according to bean variety, suggesting that bean genotype has a strong influence on the 2,5-diketopiperazines content of bean-to-bar chocolates, regardless of the degree of roasting or the technological process used by the small producers. The presence of two unique diastereoisomers in the classical chocolates bought in the supermarket indicates that the beans have probably undergone a more intense heat treatment. This study proposes the use of 2,5-diketopiperazines as potential markers of cocoa beans variety, as well as an indicator of post-harvest processing and processing technology, and highlights the potential of the molecular networks in the field of food and drink innovation as a promising tool to understand the complex chemistry of flavours.

## Graphical abstract

**Figure:**
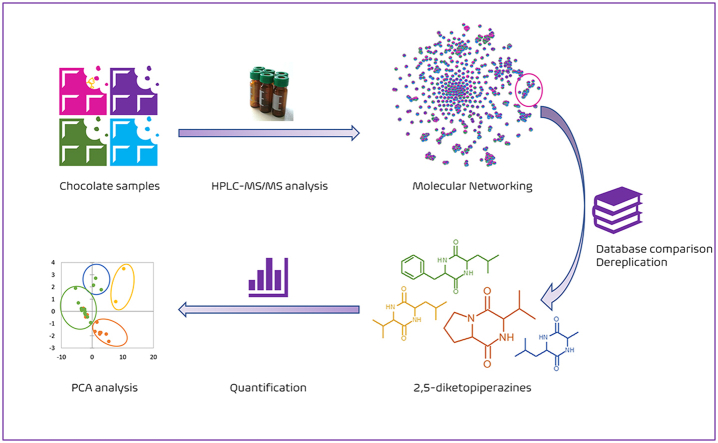

## 1 Introduction

Chocolate is a beloved product all over the world, made from the roasted beans of *Theobroma cacao*. Since approximately 1500 years B.C., when the Olmec, Maya and Aztecs civilizations drank cocoa-based beverages for religious or medicinal purposes, numerous technical innovations have occurred [1, 2]. Human taste has also evolved during this time and cocoa products are no longer consumed today for religious purposes but mainly for pleasure and its related health benefits [3]. Apart from the beloved typical chocolate aroma of chocolate candy bars, cocoa and chocolate of higher quality are also appreciated for other aromas such as floral, fruity, or nutty notes, as well as for their taste attributes such as bitterness, acidity and astringency, which are of key importance in chocolate quality [1]. Ten genetic clusters of cacao trees have been defined scientifically [4], but at trade level three main cacao varieties are used to produce chocolate: Criollo and Forastero, which form two large groups, and Trinitario, a hybrid between Criollo and Forastero [1]. While Criollo and Trinitario are usually seen as fine cocoa with fine aromatic flavours, Forastero is regarded as bulk cocoa, typically with a strong basic cocoa aroma [1, 5].

Cocoa astringency is driven by different polyphenolic compounds such as flavan-3-ols, polyphenol glycosides and N-phenylpropenoyl-*L-*amino acids, whereas acidity is driven by a few organic acids, acetic acid being the key driver in both aroma and taste [2, 6, 7]. The pleasant bitterness of chocolate is partly due to flavan-3-ols, but the key drivers of bitterness were found to be the 2,5-diketopiperazines (DKPs) together with theobromine and caffeine [8, 9].

2,5-diketopiperazines are cyclic dipeptides and were first discovered as natural products in the beginning of the 20^th^ century [10]. They are found in a large variety of food and beverages, such as beer, coffee, bread, chicken essence and Comté cheese [10]. DKPs are important sensory compounds, well-known contributors of taste in foods. Depending on their structures, they are perceived as bitter, metallic or astringent [10]. The sensorial impact of DKPs in roasted cocoa beans has been evaluated using Dose over Threshold values (DoT) [9], and ranges from 6.9 to below 0.1, depending on the structure. It is accepted that compounds with a DoT <1 have a negligeable impact on the overall taste sensation, but according to the fact that a large number of DKPs can be found in cocoa products, a synergistic effect of DKPs can be assumed considering the sum of their DoT factors [6, 11].

The roasting of the coca beans is an important step in chocolate production, well known to influence, among other things, the formation of 2,5-diketopiperazines [12, 13, 14]. However, different other steps of the transformation of cocoa beans into chocolate are responsible for the creation of those important flavour compounds. The first important step takes place during the fermentation of the cocoa beans. The proteolytic breakdown that occurs during fermentation yields hydrophilic and hydrophobic peptides and free amino acids from the cocoa storage proteins. These peptides, particularly dipeptides, and amino acids, are important precursors of chocolate flavour as they undergo cyclisation during the roasting of cocoa beans to yield not only 2,5-diketopiperazines, but also aroma compounds such as pyrazines and Strecker aldehydes [1, 15]. The content of storage protein in cocoa beans has been shown to be influenced by the genotype and the country of origin of the beans [16], which might in turn influence the content of 2,5-diketopiperazines found in chocolates [1]. Therefore, the entire transformation process of cocoa beans can influence the formation of 2,5-diketopiperazines, thereby impacting the taste and quality of the chocolate bar. More than 30 different DKPs have already been identified in cocoa products, among them *cis*-cyclo(L-Val-L-Pro) was found at the highest concentrations in every study, showing the highest dose over threshold factor for bitter taste among all other DKPs [6, 9, 12].

The detection and quantification of 2,5-diketopiperazines, which are present in cocoa beans and chocolates at relatively low levels (some μg/kg to some mg/kg), remains challenging, as their analysis using specific and sensitive detection methods such as Multiple Reaction Monitoring (MRM) techniques requires highly sensitive mass spectrometers. Therefore, 2,5-diketopiperazines are not widely analysed as markers of chocolate quality.

Molecular networking is an interesting computer-based tool to organize and visualise tandem mass spectrometry (MS^2^) data sets, becoming more and more popular in the field of natural products discovery to ease the process of dereplication (identification of already known compounds) [17, 18]. In the field of cocoa and chocolate research, molecular networks were used to identify cocoa phenolic metabolites in human urine [19] or to study the chemical composition of ruby chocolate [20]. To our knowledge, the technique of molecular networks was never employed to explore the chemical differences between cocoa beans varieties.

The principle of molecular networking is based on the assumption that similar molecules share similar fragmentation patterns, and therefore structurally related compounds are prone to form clusters within a network [21]. All 2,5-diketopiperazines share a similar fragmentation pattern, with successive characteristic losses of CO (−27.9 Da), NH_3_ (−45.0 Da) and CO again (−73.0 Da) from the parent ion, as well as characteristic immonium ions for the individual amino acids moieties formed after the cleavage of the amide bonds [22]. This characteristic allows the identification of the two amino acid moieties of diketopiperazines looking at the MS^2^ spectrum. Once identified in the molecular network, the quantification can be conducted in a second step by extracting the ion chromatogram corresponding to each diketopiperazine.

As 2,5-diketopiperazines are compounds known to be formed during the processing of cocoa beans into chocolate from precursors released during post-harvest processing, they can be considered as indicators of interest that can provide us the information of the processing of cocoa beans. The objective of the study is to use the molecular networking technique to uncover the diversity of the 2,5-diketopiperazines that can be found in different dark chocolates. The untargeted approach assesses the diversity of 2,5-diketopiperazines in different samples quite easily, to highlight the differences between chocolates samples. Therefore, a set of 33 dark chocolates from different cocoa varieties and processed by different technologies, from classical industrial chocolates where to beans can be roasted at up to 160 °C for up to 1 h [23, 24], chocolates “bean-to-bar” made by small-batch producers who control the origin of the beans and usually apply mild roasting conditions, to chocolates produced by a novel technological approach where unroasted cocoa beans are processed at low temperatures (≤60 °C) [2, 25, 26], were analysed.

## 2 Materials and methods

### 2.1 Chemicals and reagents

The following compounds were obtained commercially: cyclo(D-ala-L-Val), cyclo(-Gly-Phe), cyclo(-Leu-Phe), cyclo(-Pro-Phe) (Bachem AG, Bubendorf, Switzerland). Acetonitrile (LC-MS grade) and *n*-hexane were supplied by VWR International GmbH (Dietikon, Switzerland). Water (LC-MS grade) was supplied by Carl Roth AG (Arlesheim, Switzerland), ethyl acetate and methanol (LC-MS grade) by Sigma-Aldrich (Merck AG, Zug, Switzerland). The description of the different chocolates analysed for this study is given in Table 1. Classic chocolates are defined in this paper as mass market/industrial chocolates produced by big manufacturers, cold extraction chocolates are defined as chocolates produced by a new patented process from unroasted fermented beans [25, 26], and bean to bar chocolates are commonly understood as chocolates produced by small batch producers from cocoa beans of known origin. All chocolates were purchased from local stores in Switzerland. The reference chocolates were produced with cocoa beans of defined varieties and provided by the Cocoa of Excellence program [27]. The country of origin, the cocoa variety and the roasting parameters of the beans (temperature and time) mentioned by the manufacturers are reported in Table 1.

**Table 1:** Description of the different chocolates analysed for the study.

Fabrication method	% cacao	Origin of the beans (Region, Country)	Cocoa variety	Code	Roasting temperature (°C)	Roasting time (min)
Classic	70 %	Not communicated	Not communicated	CL1	NC∗	NC
Classic	72 %	Not communicated	Not communicated	CL2	NC	NC
Classic	85 %	Not communicated	Not communicated	CL3	NC	NC
Cold extraction	70 %	Caribbean (Cuba)	Trinitario	SB1	0	0
Cold extraction	75 %	South America (Guatemala)	Trinitario/Criollo	SB2	0	0
Cold extraction	80 %	South America (Peru)	Criollo	SB3	0	0
Small Batch/Bean to Bar	70 %	South America (Colombia)	Criollo	SB4	116	35
Small Batch/Bean to Bar	81 %	South America (Colombia)	Criollo	SB5	120	22
Small Batch/Bean to Bar	80 %	South America (Colombia)	Criollo	SB6	118	23
Small Batch/Bean to Bar	70 %	Central America (Mexico)	Trinitario/Criollo	SB7	118	32
Small Batch/Bean to Bar	76 %	Central America (Mexico)	Criollo	SB8	NC	NC
Small Batch/Bean to Bar	70 %	Central America (Mexico)	Criollo	SB9	112	35
Small Batch/Bean to Bar	70 %	South America (Mexico)	Criollo	SB10	118	23
Small Batch/Bean to Bar	70 %	Africa (Madagascar)	Trinitario	SB11	114	28
Small Batch/Bean to Bar	70 %	Africa (Madagascar)	Trinitario	SB12	NC	NC
Small Batch/Bean to Bar	75 %	Africa (Madagascar)	Trinitario	SB13	NC	NC
Small Batch/Bean to Bar	75 %	Africa (Madagascar)	Trinitario	SB14	106	52
Small Batch/Bean to Bar	72 %	Africa (Madagascar)	Trinitario	SB15	NC	NC
Small Batch/Bean to Bar	82 %	Africa (Madagascar)	Trinitario/Criollo	SB16	NC	NC
Small Batch/Bean to Bar	70 %	Africa (Tanzania)	Trinitario	SB17	117	30
Small Batch/Bean to Bar	75 %	Africa (Tanzania)	Trinitario	SB18	NC	NC
Small Batch/Bean to Bar	72 %	Africa (Tanzania)	Trinitario	SB19	NC	NC
Small Batch/Bean to Bar	75 %	Africa (Tanzania)	Trinitario	SB20	NC	NC
Small Batch/Bean to Bar	72 %	Africa (Tanzania)	Trinitario	SB21	NC	NC
Small Batch/Bean to Bar	70 %	West Africa (Congo)	Forastero	SB22	NC	NC
Small Batch/Bean to Bar	75 %	West Africa (Ghana)	Forastero	SB23	115	23
Small Batch/Bean to Bar	60 %	West Africa (Ghana)	Forastero	SB24	NC	NC
Small Batch/Bean to Bar	70 %	West Africa (Cameroon)	Trinitario	SB25	122	22
Small Batch/Bean to Bar	75 %	West Africa (Ivory Coast)	Not communicated	SB26	NC	NC
CoEx Reference	75 %	Not communicated	Forastero	Ref1	129	24
CoEx Reference	75 %	Not communicated	Criollo	Ref2	110	25
CoEx Reference	75 %	Not communicated	Trinitario	Ref3	119	24
CoEx Reference	75 %	Not communicated	Trinitario	Ref4	119	23

∗ NC: not communicated by the manufacturer.

### 2.2 Extraction

The defatting of the samples was performed according to Pedan et al. [28], whereby 50 g of chocolate were used for sample preparation. First, chocolate samples were crushed in an Ika A11 basic analytical mill before being lyophilized (Martin Christ GmbH, Osterode am Harz, Germany). The samples were extracted with *n*-hexane at a ratio 1:5 (w/v) for 8 min at room temperature. The mixture was then centrifuged for 2 min at 4400 rpm (Eppendorf model 5702, Schönenbuch, Switzerland), the *n*-hexane was removed, and the powder was extracted six times in total, before being freeze-dried to remove any trace of water and organic solvent.

0.4 g of defatted chocolate powder was extracted with 2 mL of ethyl acetate for 2 h at room temperature under constant shaking using an overhead shaker (Hettich Labtechnology, Tuttlingen, Germany) (modified from André et al. [29]). The mixture was then centrifuged for 15 min at 4400 rpm (Eppendorf model 5702, Schönenbuch, Switzerland) and the supernatant was carefully collected using a glass pipette. The ethyl acetate was evaporated under a gentle nitrogen flux at 35 °C. The dried extract was reconstituted in 1 mL of a 90:10 methanol: water solution before being cleaned from traces of lipophilic compounds using SPE. SPE cartridges (Sep-Pak C18 Vac 1cc (100mg) cartridges, Waters AG, Baden, Switzerland) were conditioned with 1 mL methanol followed by 1 mL 90:10 methanol water. 1 mL of the extract was loaded onto the column and the molecules of interest were eluted with 1 mL of 90:10 methanol:water. The eluted samples were analysed using HPLC-MS/MS. The extractions were made in triplicates for each chocolate.

For quantification, the method of Stark and Hofmann was used [9], with some modifications: defatted cocoa powder (0.4 g) was spiked with the internal standard *trans*-cyclo(D-ala-L-val) and cyclo(-gly-phe) dissolved in methanol. The spiked powder was allowed to equilibrate for 1 h under the chemical hood before being extracted as described above. 2,5-diketopiperazines containing the aromatic amino acids phenylalanine or tyrosine were quantified by using cyclo(-gly-phe) as the internal standard, as this compound was not present in the samples analysed. The other 2,5-diketopiperazines were quantified using *trans*-cyclo(D-ala-L-val) as the internal standard. The amount of the individual 2,5-diketopiprazines was calculated using response curves that had been determined by analysing solutions containing defined amounts of the internal standards and the target compounds in different concentrations (Figures S1 and S2). The results are expressed as mg/kg of defatted material.

### 2.3 HPLC-MS/MS analysis

LC-MS/MS analyses were conducted on a system consisting of an Agilent 1290 Infinity II chromatographic system coupled to an Agilent 6530 Q-TOF mass spectrometer. Separation of analytes was performed using an Agilent Poroshell 120 EC-C18 (2.1 × 150 mm, 2.7 μm) column protected by a guard column (Agilent EC-18, 2.1 × 5 mm, 2.7 μm). The flow rate was set to 0.25 mL/min, and the column temperature set at 40 °C. The two elution mobile phases were made up of water + 0.1% formic acid (FA) (mobile phase A) and acetonitrile + 0.1% FA (mobile phase B). Gradient elution was as follows: 0–5 min, 5% B; 35 min, 60% B; 45 min, 100% B; 55 min, 100% B; 56 min, 5% B. Re-equilibration time was 8 min. Injection volume was 3.5 μL.

The MS analyses were performed using Agilent 6530 Q-TOF instrument in positive ionisation mode (ESI), in the spectral range of 100–1000 Da. Nitrogen served as the nebuliser and collision gas. The MS parameters were as follows: gas temperature, 350 °C; drying gas, 10 L/min; nebuliser, 35 psi; sheath gas temperature, 375 °C; sheath gas flow, 11 L/min; capillary voltage, 4000 V; fragmentor voltage, 130 V. For MS/MS analysis, the collision energy used was 25 eV using Agilent auto MS/MS mode.

### 2.4 Molecular network construction: MZmine 2 data preprocessing parameters

The MSconvert software (v. 3.0) was used to convert all the d (Agilent standard data format) MS^2^ data files to mzXML format [30]. All mzXML files were then processed using MZmine 2.53 [31]. The mass detection was realized keeping the noise at 0. The chromatogram building was achieved using the ADAP chromatogram builder with a minimum group size of 4 scans, a group intensity threshold of 3.0E3, a minimum highest intensity of 4.0E3 and a *m/z* tolerance of 0.005 *m/z* (or 20 ppm) [32]. The chromatogram deconvolution algorithm ADAP wavelets was used with the following settings: S/N threshold = 8, minimum feature height: 4000, coefficient/area threshold = 15, peak duration range = 0.05–2.00, RT wavelet range = 0.01–0.10, *m/z* range for MS2 scan paring = 0.02, RT range for MS^2^ scan pairing = 0.5. Chromatograms were deisotoped using the isotopic peaks grouper algorithm with an *m/z* tolerance of 0.005 or 20 ppm, and an RT tolerance of 0.2 min with a maximum charge of 2. Peak alignment was performed using the join aligner method with the following parameters: *m/z* tolerance = 0.004 or 10 ppm, weight for *m/z* = 2, absolute RT tolerance = 0.5 min, weight for RT = 1. The peak list was gap filled with the same RT and *m/z* range gap filler module (*m/z* tolerance at 0.004 or 10 ppm), and finally a reset of the peak number ID was done using the feature list row filter module. The mgf spectral data file and its corresponding csv metadata file containing retention times and peak areas were exported using the “Export to GNPS” module.

### 2.5 Molecular network visualisation

Importing the mgf and csv files to the MetGem software (v. 1.3.6), a molecular network was created [21]. The data was filtered by removing all MS/MS peaks within +/– 17 Da of the precursor *m/z*. MS/MS spectra were window filtered by choosing only the top 6 peaks within the ±50 Da window throughout the spectrum. Precursor ion mass tolerance and fragment ion mass tolerance were set at 0.02 Da. Consensus spectra that contained less than 1 spectrum were discarded. A network was then created where edges were filtered to have a cosine score above 0.65 and more than 4 matched peaks. Further edges between two nodes were kept in the network if, and only if, each of the nodes appeared in each other's respective top 10 most similar nodes. For the colour mapping process, new columns were created in MetGem using the “mean()” function in order to calculate the average extracted ion chromatogram area for each bean variety. Those columns were used to colour map each node with a pie-diagram where the proportions are based on the average extracted ion chromatogram areas (EIC/XIC) of each cocoa variety.

### 2.6 Statistical analysis

Data analysis was done using the XLSTAT statistical and data analysis solution for Excel (Premium edition 2022.2.1). Principal component analysis (PCA) was applied to assess the differences between all the samples and detect 2,5-diketopiperazines that best discriminate between chocolate samples. Pearson’s correlation coefficient was calculated using a p-value of 5%.

## 3 Results

The chocolate extracts were analysed by HPLC-MS/MS. The data collected were used to build a molecular network using the MetGem software [21]. Based on the hypothesis that chemically similar compounds will share similar fragmentation pathways and therefore related neutral fragments and losses, the molecular networking technique creates a graphical representation of one or several samples. Each node on the molecular network represents one ion found within at least one sample. The closer two nodes are on the network, the closer they are in terms of chemical structure [21].

**Figure 1:**
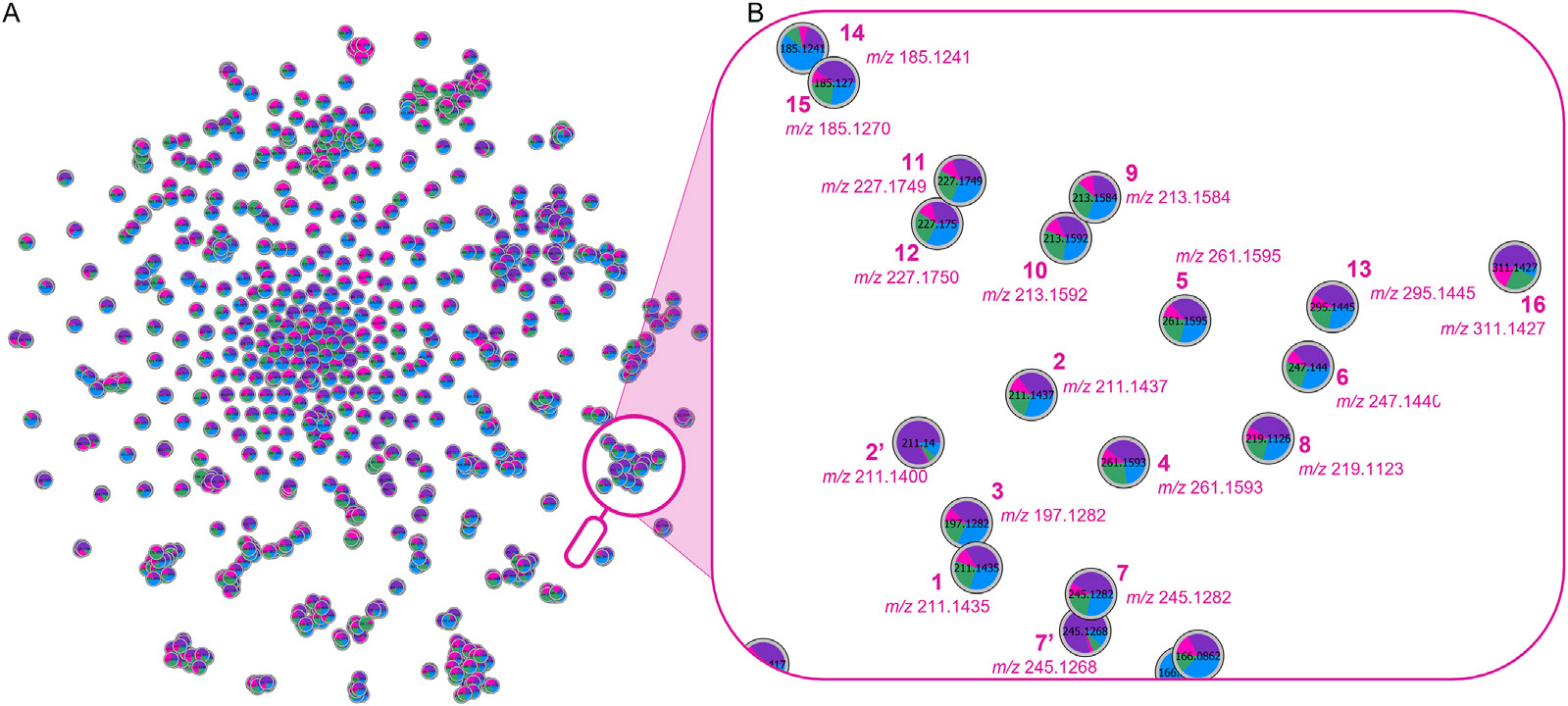
Molecular network (t-SNE output) built with the chocolate dataset (A) with a focus on the diketopiperazines cluster (B). Relative quantification values of each ion within the extracts are represented as an average of XIC area-dependent pie chart. Color mapping correspond to the cocoa variety (pink: Criollo; green: Trinitario; blue: Forastero; purple: unknown variety).

The t-SNE based representation of the molecular network is given in Figure 1 and enables the visualisation of the molecular diversity of the different chocolate samples. The comparison of the MS^2^ spectra of the chocolate extracts with online MS^2^ spectral libraries allowed the identification of 4 diketopiperazines (cyclo(leu-pro), cyclo(phe-pro), cyclo(val-pro) and cyclo(phe-leu)) within the network. By studying the closest nodes around those four diketopiperazines, notably their *m/z* and MS^2^ spectra, 14 other diketopiperazines were identified within the chocolate extracts. The dereplication of the 14 diketopiperazines was based on their typical fragmentation pattern consisting of successive characteristic losses of CO (−27.9 Da), NH_3_ (−45.0 Da) and CO again (−73.0 Da) from the parent ion, as well as characteristic fragment ions (immonium ions) of amino acids residues formed after the cleavage of the amide bonds [22]. An example of a characteristic MS^2^ spectra is given in the supplementary material in Figure S4, and all HPLC-MS/MS data of the identified compounds are given in Table S1.

Although it was possible to observe several isomeric peaks of some compounds, the 2,5-diketopiperazines in this investigation do not have an absolute stereochemical assignment. Based on the literature, the major diastereoisomers were assigned as *cis*-isomers [3, 4]. Moreover, it was possible to distinguish between leucine and isoleucine residues when looking at the MS^2^ data: 2,5-diketopiperazines with a leucine moiety were characterized by the presence of two product ions at *m/z* 86.09 and 72.04, when 2,5-diketopiperazines with an isoleucine moiety were characterized by the presence of the two product ions at *m/z* 86.09 and 69.07 (Table S1) [33]. All MS^2^ spectra are available in the supplementary material (Figure S1–S16).

The nodes in the molecular network (Figure 1) are coloured according to the cocoa variety to easily spot the differences between the samples. The colour group mapping in Figure 1 (pink for Criollo, green for Trinitario; blue for Forastero; purple for unknown variety) is made in the form of a pie-chart. The proportion of each colour on the pie-chart represent the average peak area of the compound in the different cocoa varieties. Looking at the 2,5-diketopiperazines cluster presented in Figure 1B, conclusions about the DKP composition of the different samples can be drawn even before the quantification step. As indicated by the smaller proportion of the pink in the nodes’ pie-charts, the Criollo variety seems to contain lower quantities of all DKPs identified. On the other hand, chocolates made with beans of unknown variety and Forastero type (violet and blue respectively, in Figure 1) seems to contain higher quantities of DKPs according to the colour mapping. Moreover, two nodes, which were identified as diastereoisomers of cyclo(pro-leu) (**2’**) and cyclo(phe-pro) (**7’**) are predominantly coloured in purple (Figure 1B). The colour mapping indicates that they are mainly present in the chocolates of unknown variety. Therefore, the study of the molecular network allows a rapid first analysis of a broad dataset, allowing differences between Criollo and Forastero/Unknown origin samples to be pinpointed.

**Figure 2:**
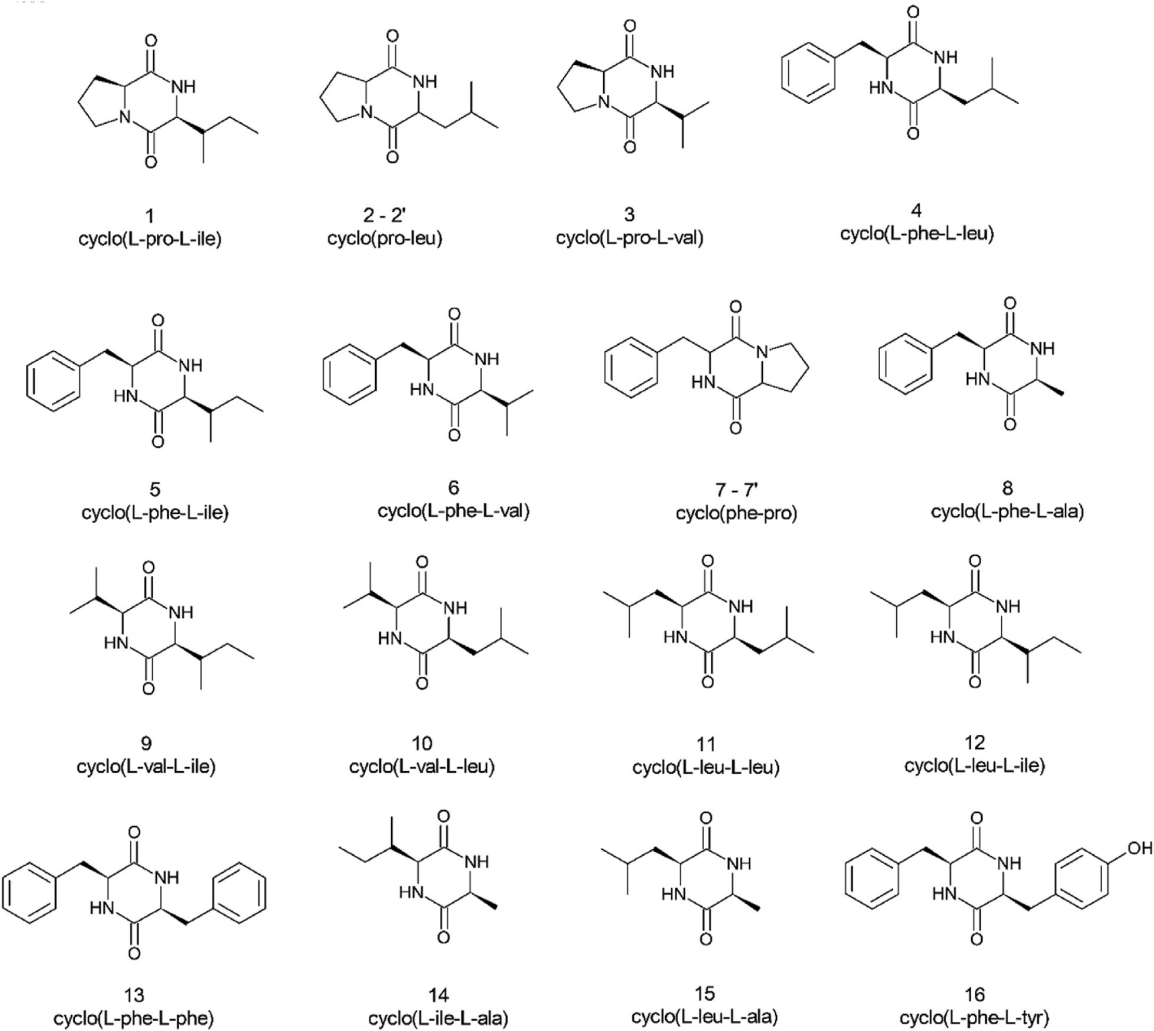
Structures of the 2,5-diketopiperazines identified in the chocolate samples.

Overall, 18 diketopiperazines were found in the chocolate extracts: cyclo(L-pro-L-ile) (**1**), two diastereoisomers of cyclo(pro-leu) (**2**-**2’**), cyclo(L-pro-L-val) (**3**), cyclo(L-phe-L-leu) (**4**), cyclo(L-phe-L-ile) (**5**), cyclo(L-phe-L-val) (**6**), two diastereoisomers of cyclo(phe-pro) (**7**-**7’**), cyclo(L-phe-L-ala) (**8**), cyclo(L-val-L-ile) (**9**), cyclo(L-val-L-leu) (**10**), cyclo(L-leu-L-leu) (**11**), cyclo(L-leu-L-ile) (**12**), cyclo(L-phe-L-phe) (**13**), cyclo(L-ile-L-ala) (**14**), cyclo(L-leu-L-ala) (**15**) and cyclo(L-phe-L-tyr) (**16**) whose structures are represented in Figure 2.

**Figure 3:**
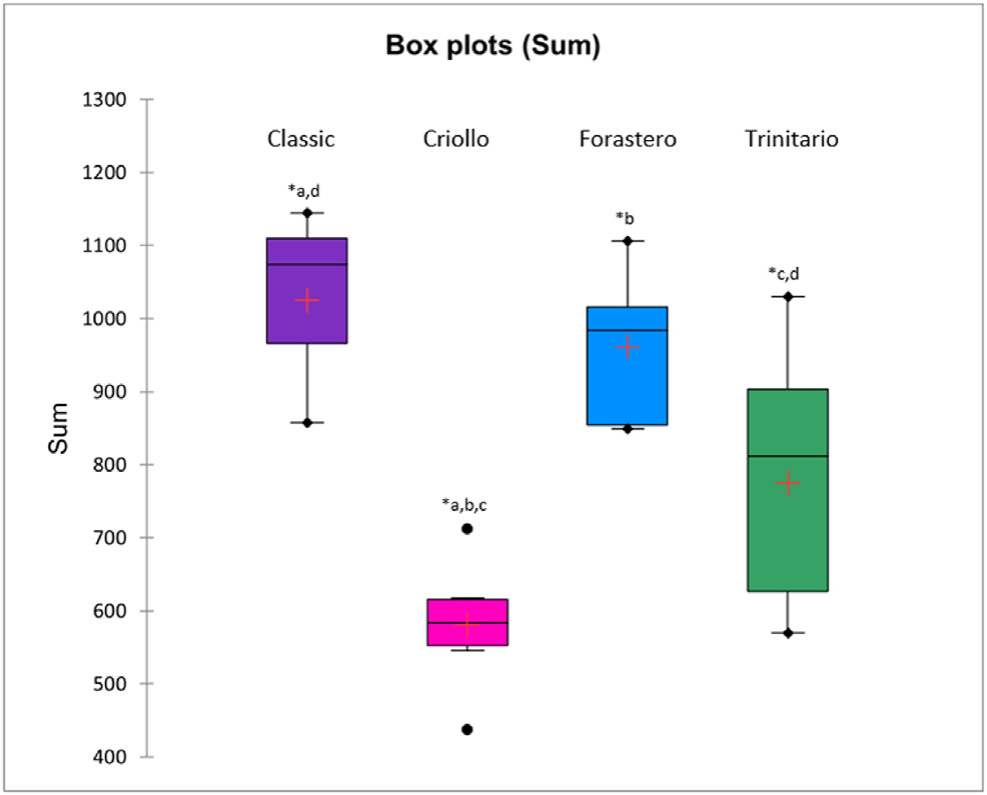
Box plots of the total 2,5-diketopiperazines content (mg/kg) in chocolates made with different bean varieties; ∗ = P < 0.05 (t-test).

To confirm the deductions made based on the molecular network visualization, the different diketopiperazines were quantified by extracting the ion chromatogram (EIC) for each compound and using two internal standards, namely cyclo(D-ala-L-val) and cyclo(-gly-phe). The quantification of each chocolate sample is presented in Table S2. As shown in Figure 3, Criollo chocolates have a significant lower total content in 2,5-diketopiperazines (∗a,b,c; *p* < 0.05) compared to all other cocoa varieties. The three classic chocolates made with beans of unknown variety have a significantly higher total content in 2,5-diketopiperazines (*p* < 0.05) compared to Criollo and Trinitario varieties, with an average total DKP content of 1025 mg/kg for the classic chocolate samples, when the average of the Criollo and Trinitario samples is 581 and 775 mg/kg respectively (∗c,d in Figure 3).

Looking more specifically into the composition, cyclo(L-pro-L-val) (**3**) was found to be the most abundant 2,5-diketopiperazine in all samples and particularly in the classic chocolates where its quantity was found to be higher than 250 mg/kg, as already reported in the literature [9]. Cyclo(L-pro-L-leu) (**2**), cyclo(L-leu-L-val) (**10**) and cyclo (L-leu-L-leu) (**11**) were the 3 others most important 2,5-diketopiperazines found in all samples. The diastereoisomers cyclo(pro-leu) (**2’**) and cyclo(phe-pro) (**7’**) were only quantifiable in the three classic chocolate samples, which confirm findings highlighted by the pie-chart colour mapping in the molecular network (Figure 1B). To our knowledge, cyclo(L-ile-L-val) (**9**), cyclo(L-leu-L-ile) (**12**) and cyclo(L-phe-L-phe) (**13**) were detected here for the first time in chocolates.

**Figure 4:**
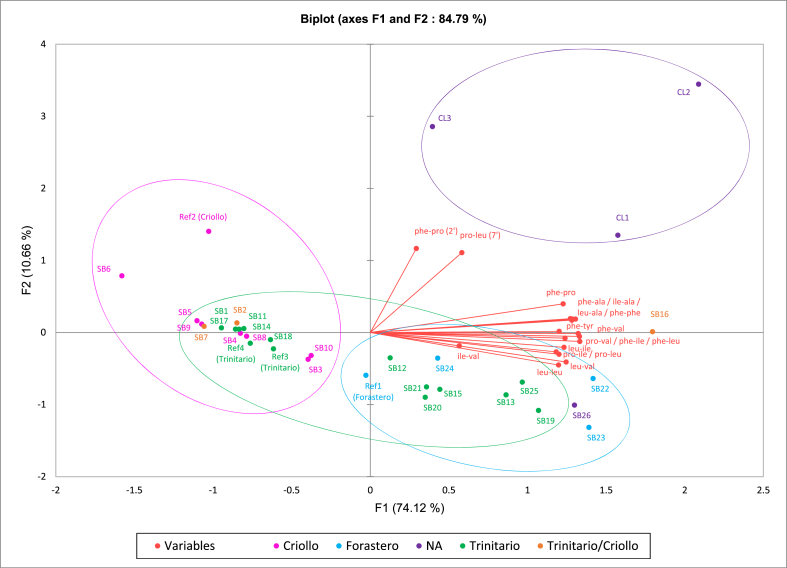
Biplot of the first two principal components of the principal component analysis (PCA) of the 2,5-diketopiperazines profiles of the different chocolates. Colours represent the cocoa variety: pink Criollo; green Trinitario; blue Forastero, and purple unknown origin. The samples with a mix of Trinitario/Criollo are colored in orange. Chocolate codes are given in Table 1 (CL: Classic, SB: Small Batch, Ref: Reference).

To better assess the differences in the 2,5-diketopiperazines profiles of the different chocolates, a principal component analysis (PCA) was performed (Figure 4).

In Figure 4, the two first components of the analysis explain 84.79 % of the variance. Four clusters are formed within the PC1 and PC2 axes. Interestingly, three separated clusters can be observed on the PCA biplot in Figure 4, corresponding to different cocoa varieties: Forastero in blue, Criollo in pink and the classical chocolate made with beans from unknown origin in purple, and thus independently of the roasting process used by the different chocolate producers. The Trinitario samples are found to be distributed inside Criollo and Forastero clusters. All reference chocolates grouped with chocolates made with the same cocoa variety.

The Forastero cluster in blue is characterized by a significantly higher total amount of 2,5-diketopiperazines (between 848 and 1106 mg/kg) compared to the Criollo cluster (between 438 and 712 mg/kg) (t-test, *p* < 0.01). The samples belonging to this cluster are characterized by the presence of cyclo(L-pro-L-val) (**3**), cyclo(L-leu-L-val) (**10**), cyclo(L-leu-L-leu) (**11**) and cyclo(L-phe-L-tyr) (**16**) in higher amounts than in the chocolates made with Criollo variety (t-test, *p* < 0.01).

In the Criollo cluster (circled in pink in Figure 4), the chocolates were characterized by the lowest quantities of all diketopiperazines detected. Particularly, cyclo(L-phe-L-tyr) (**16**) was not quantifiable because of its too low amounts in those chocolates.

The three classic chocolates made with beans of unknown origin form the last cluster (circled in purple in Figure 4) characterized by a high content of 2,5-diketopiperazines, not significantly different from the average content of Forastero chocolates, and by the presence of both diastereoisomers of cyclo(-pro-leu) (**2’**) and cyclo(-phe-pro) (**7’**), as well as the presence of cyclo(L-phe-L-tyr) (**16**).

Interestingly, the Trinitario samples are spread among the Forastero and Criollo clusters. Four chocolates (SB11, SB14, SB17, SB18) and the two Trinitario reference chocolates show a DKP profile comparable to the one of Criollo samples with an overall lower level of total DKPs, except for the presence of little amounts of cyclo(L-phe-L-tyr) (**16**). The rest of the Trinitario chocolates (SB12, SB13, SB15, SB19, SB20, SB21 and SB25) show a DKP profile similar to the one of the Forastero samples, in particular with a higher content of total DKPs.

Finally, the chocolates produced by the cold extraction process (SB1, SB2, SB3) where no roasting of the beans takes place, are found in the Criollo cluster, and shows no significant difference in terms of 2,5-diketopiperazine content or profile with the other chocolate samples in this cluster that underwent a roasting step. This indicates that the lower temperatures of the new technological process (<60 °C) were still sufficient to generate 2,5-diketopiperazines, in the same amounts as in cocoa beans of the same varieties roasted at higher temperatures (>100 °C), highlighting the influence of the beans’ variety in 2,5-diketopiperazines formation.

**Figure 5:**
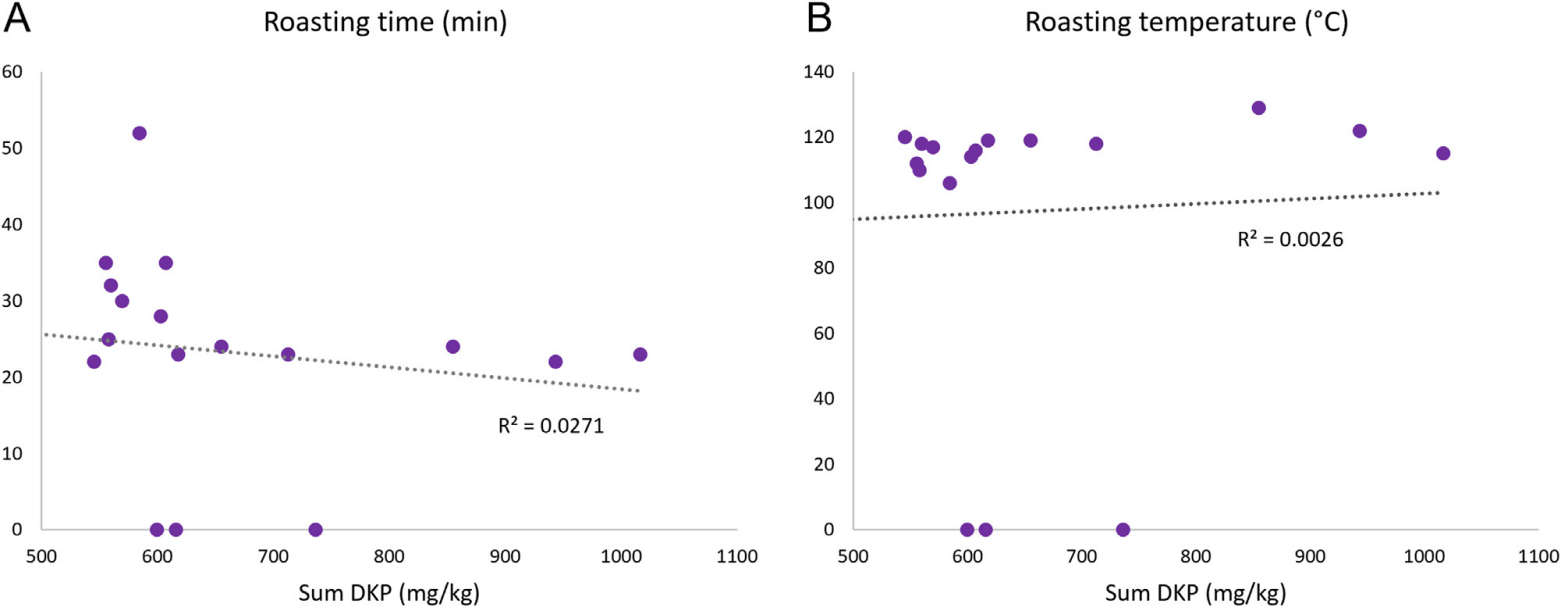
Correlation between A. Roasting time (min) and total DKPs (mg/kg); B. Roasting temperature (°C) and total DKP content (mg/kg).

To verify the hypothesis that the amount of 2,5-diketopiperazines found in the chocolate samples is independent of the roasting parameters, Pearson’s correlation test was conducted using the roasting data of 18 samples of the dataset. The R^2^ factors obtained were respectively 0.0026 (roasting temperature) and 0.0271 (roasting duration) (Figure 5).

## 4 Discussion

The differences found in terms of 2,5-diketopiperazines profiles between the chocolates and the clustering of the cocoa varieties can be explained by different factors. Despite the undeniable importance of post-harvest processes in the development of flavours in cocoa beans, the bean variety has been shown to also influence the chemical composition, in particular the type and quality of bean storage proteins, polyphenols, or polysaccharides, which are degraded during fermentation and drying to form flavour precursors [16, 34, 35]. Kumari et al. observed in their study that the bean genotype as well as the geographical origin of the bean influence the storage protein content [16]. As cacao storage proteins generate the precursors of 2,5-diketopiperazines (dipeptides, amino acids) during fermentation and drying [16], a lower protein content in unfermented beans would logically lead to a lower amount of 2,5-diketopiperazines created during the chocolate processing. The different traditional fermentation protocols used by the cocoa farmers in South America, West Africa, and other African countries like Madagascar and Tanzania also influence the formation of those precursors. For example, Criollo is traditionally fermented for a shorter period than Forastero (2–3 days for Criollo versus 5–8 days for Forastero) in order to obtain an optimal flavour profile [1, 34]. Indeed, fine flavour aromas seems to be reduced in intensity with increasing fermentation times [1]. Therefore, it can be logically assumed that the traditionally longer fermentation time observed for the Forastero variety could lead to a higher release of precursors that will be converted to 2,5-diketopiperazines at a later stage during chocolate making process.

The results of this study indicate that the beans variety has a strong influence on the DKP content of chocolates, regardless of the degree of roasting used by the small-batch producers. Our analysis reveals that the chocolates made of Criollo beans do contain lower levels of 2,5-diketopiperazines than the chocolates made of Forastero beans, thus independently of the roasting parameters (temperature or duration) used by the producers, as statistically tested using the Pearson’s correlation coefficient. The Criollo variety is known to produce mild, less bitter and less astringent chocolates, usually called fine flavour chocolates, in comparison to Forastero variety mainly cultivated to produce bulk chocolate [1]. The lower 2,5-diketopiperazines content found in the chocolates made with Criollo variety is thus in line with this characteristic.

The distribution of the Trinitario samples in both the Criollo and Forastero clusters can also be explained looking at the fact that the Trinitario variety originates from a cross between Criollo and Forastero. It has been distributed worldwide since 1880 and its aromatic profile depends on whether the Criollo or the Forastero background is predominant but is classified as fine flavour cocoa after Criollo [1]. Therefore, the Trinitario samples found within the Criollo cluster in the PCA (Figure 4) are thought to express predominantly their Criollo background, which is supported by the fact that they contain low levels of 2,5-diketopiperazines as do the Criollo chocolates. On the other hand, the Trinitario samples found in the Forastero cluster are thought to express predominantly their Forastero background (more bitter and astringent) [36], as supported by their higher content of 2,5-diketopiperazines.

Interestingly, the three chocolates produced with the cold extraction process contain the same amount of DKPs as the samples from the Criollo cluster. As the technological process of production is based on the cold extraction of fermented beans without any roasting step, a low level of DKPs was expected in the chocolates. This finding indicates that although the beans were not roasted, the process steps where heat is applied (<60 °C) also allows the transformation of the free amino acids precursors into 2,5-diketopiperazines [2, 25, 26], in the same amount as samples sharing similar genetic backgrounds.

While the influence of the roasting procedure on the 2,5-diketopiperazines profiles was not observed in the bean-to-bar chocolates, this influence can be observed in the classic chocolates analysed. In comparison with the bean-to-bar chocolates produced by small batch producers and the reference chocolates analysed, the classical chocolates contain two unique 2,5-diketopiperazines, namely diastereoisomers of cyclo(pro-leu) and cyclo(phe-pro). Epimerisation is a common phenomenon occurring during cocoa roasting when heating diketopiperazines in a non-neutral pH [12, 37]. The increased presence of minor isomers with increased temperatures (150 °C vs 120 °C) and increased times was observed by Andruszkiewicz et al [12]. Therefore, the unique presence of the isomers of cyclo(pro-leu) and cyclo(phe-pro) in the classic chocolates can be seen as an indicator of a higher roasting temperature and/or a higher roasting time used by the producers of mass-market chocolates, in comparison with small batch producers.

## 5 Conclusions

The use of molecular networking offers several advantages compared to the targeted approach usually employed to quantify 2,5-diketopiperazines in cocoa samples. First, the molecular networking technique is based on an untargeted analysis, therefore allowing the chemical diversity of the samples to be visualized and not only to focus on pre-defined compounds. Second, the organization of the complex MS^2^ data in a graphic form where structurally similar compounds are close to each other, together with the possibility to compare automatically MS^2^ spectra with a growing open databank, ease the identification of compounds of interest by simplifying the dereplication process. Moreover, the possibility to apply a colour mapping for each ion in the form of a pie-chart diagram allows differences between samples to be clearly visualized before proceeding to quantification. The colour mapping also highlights compounds found only in some samples. Altogether, molecular networking is a valuable tool to visualize the chemical diversity of a large number of samples and highlight early in the screening process interesting differences between samples that can be further studied with a quantification step.

This approach led us to identify 18 different 2,5-diketopiperazines within the 33 chocolates analysed. Cyclo(L-pro-L-val) was found as the major 2,5-diketopiperazine in all samples, followed by cyclo(L-pro-L-leu), cyclo(L-leu-L-val) and cyclo (L-leu-L-leu). The majority of the 2,5-diketopiperazines were found in all chocolates, but two diastereoisomers of cyclo(-pro-leu) and cyclo(-pro-phe) as well as cyclo(L-phe-L-tyr) were found only in some samples. To the best of our knowledge, this study also reports for the first time the presence of cyclo(L-ile-L-val), cyclo(L-leu-L-ile) and cyclo(L-phe-L-phe) in chocolates.

A principal component analysis reveals the clustering of the 33 chocolates according to the variety of the beans. The results highlight that Criollo chocolates contain lower amounts of 2,5-diketopiperazines, and Forastero chocolates higher amounts, and this irrespective of the roasting parameters used by the small batch producers. These findings correlate with the differences in protein content in unfermented beans from different origins described earlier [16], as well as the different fermentation process traditionally used and are in line with the milder, less bitter and less astringent character of Criollo chocolates [1, 34].

Therefore, the findings of the study indicate that the bean variety influence greatly the amount of 2,5-diketopiperazines found in bean-to-bar chocolates, irrespective of the processing technology and mild roasting process used by the small-batch producers. However, the presence of DKPs isomers in the classic chocolates analysed indicate that the industrial processing of beans was conducted under higher roasting conditions (temperature and/or time). For that reason, 2,5-diketopiperazines could be used as markers of cocoa bean variety and more generally as an indicator of harsh cocoa beans processing. A further study, where the beans could be analysed from the cocoa farm all along the transformation process to the chocolate bar would facilitate confirmation of this finding. In addition, this study highlights the potential of the molecular networks in the field of food and drink innovation, as a promising tool to understand the complex chemistry of taste and aroma.

## Declarations

### Author contribution statement

Amandine André: Conceived and designed the experiments; Performed the experiments; Analyzed and interpreted the data; Contributed reagents, materials, analysis tools or data; Wrote the paper.

Lisa Ullrich; Bettina Casty: Performed the experiments.

Irene Chetschik: Analyzed and interpreted the data; Contributed reagents, materials, analysis tools or data; Wrote the paper.

### Funding statement

This research was funded by the ZHAW Department N internal funding 2020 (grant number 9710.3.15.5.0344.01).

### Data availability statement

Data included in article/supp. material/referenced in article.

### Declaration of interest’s statement

The authors declare no conflict of interest.

### Additional information

No additional information is available for this paper.

## Appendix A Supplementary data

Supplementary_revised Supplementary_revised
