# Supplementary material for: Use of molecular networking to identify 2,5-diketopiperazines in chocolates as potential markers of bean variety
Source: Heliyon. 2022 Sep 27;8(9):e10770. doi: 10.1016/j.heliyon.2022.e10770 (PMC9525904; doi:10.1016/j.heliyon.2022.e10770)
Supplement: Supplementary_revised [file mmc1.pdf]

Use of Molecular Networking to identify 2,5-diketopiperazines in chocolates as potential markers of bean variety

Amandine André, Bettina Casty, Lisa Ullrich and Irene Chetschik *

ZHAW Zurich University of Applied Sciences, School of Life Sciences and Facility Management, ILGI Institute of Food and Beverage Innovation, Research group Food Chemistry, 8820 Wädenswil, Switzerland.

* Correspondence: irene.chetschik@zhaw.ch (I.C)

Supplementary Material

Table S 1. HPLC-MS/MS data of diketopiperazines identified in chocolate samples and prediction of their amino acid composition 2
Table S 2. Quantification of 2,5-diketopiperazines in 33 chocolate samples expressed as mg of diketopiperazine per kg of defatted chocolate powder 3

Figure S 1. Quantification of selected diketopiperazines using trans-cyclo(D-ala-L-val) as internal standard (IS) . 4
Figure S 2. Quantification of selected diketopiperazines using cyclo(-gly-phe) as internal standard (IS). 5
Figure S 3. MS/MS spectra of cyclo(L-pro-L-ile) **1** 6
Figure S 4. MS/MS spectra of cyclo(-pro-leu) **2** 6
Figure S 5. MS/MS spectra of cyclo(-pro-leu) **2'** 6
Figure S 6. MS/MS spectra of cyclo(L-pro-L-val) **3** 7
Figure S 7. MS/MS spectra of cyclo(L-phe-L-leu) **4** 7
Figure S 8. MS/MS spectra of cyclo(L-phe-L-ile) **5** 7
Figure S 9. MS/MS spectra of cyclo(L-phe-L-val) **6** 8
Figure S 10. MS/MS spectra of cyclo(-phe-pro) **7** 8
Figure S 11. MS/MS spectra of cyclo(-phe-pro) **7'** 8
Figure S 12. MS/MS spectra of cyclo(L-phe-L-ala) **8** 9
Figure S 13. MS/MS spectra of cyclo(L-val-L-ile) **9** 9
Figure S 14. MS/MS spectra of cyclo(L-val-L-leu) **10** 9
Figure S 15. MS/MS spectra of cyclo(L-leu-L-leu) **11** 10
Figure S 16. MS/MS spectra of cyclo(L-leu-L-ile) **12** 10
Figure S 17. MS/MS spectra of cyclo(L-phe-L-phe) **13** 10
Figure S 18. MS/MS spectra of cyclo(L-ile-L-ala) **14** 11
Figure S 19. MS/MS spectra of cyclo(L-leu-L-ala) **15** 11
Figure S 20. MS/MS spectra of cyclo(L-phe-L-tyr) **16** 11

Table S 1. HPLC-MS/MS data of diketopiperazines identified in chocolate samples and prediction of their amino acid composition

Number	Compound	Chemical formula	Average RT (min)	$[M+H]^+$	MS/MS product ions				
					$[M+H-CO]^+$	$[M+H-HCONH_2]^+$	$[M+H-CO-HCONH_2]^+$	Fragment(s) ion(s) amino acid 1	Fragment(s) ion(s) amino acid 2
1	Cyclo(L-pro-L-ile)	$C_{11}H_{18}N_2O_2$	16.52	211.1437	183.1517	*n.d.*	138.1286	70.0658 (pro)	86.0961 ; 69.0717 (ile)
2	Cyclo(-pro-leu)	$C_{11}H_{18}N_2O_2$	17.40	211.1437	*n.d.*	*n.d.*	138.1266	70.0655 (pro)	86.0964 ; 72.0476 (leu)
2’	Cyclo(-pro-leu)	$C_{11}H_{18}N_2O_2$	17.03	211.1437	183.1487	*n.d.*	138.1211	70.0647 (pro)	86.0966 ; 72.0451 (leu)
3	Cyclo(L-pro-L-val)	$C_{10}H_{16}N_2O_2$	12.22	197.1287	169.1279	*n.d.*	124.1079	70.0654 (pro)	72.0804 (val)
4	Cyclo(L-phe-L-leu)	$C_{15}H_{20}N_2O_2$	23.20	261.1596	233.1646	216.1292	188.1393	120.0800 (phe)	86.0968 ; 72.0388 (leu)
5	Cyclo(L-phe-L-ile)	$C_{15}H_{20}N_2O_2$	23.46	261.1596	233.1588	*n.d.*	188.1438	120.0802 (phe)	86.0982 ; 69.0679 (ile)
6	Cyclo(L-phe-L-val)	$C_{14}H_{18}N_2O_2$	21.18	247.1444	219.2991	202.1178	174.1274	72.0797 (val)	120.0802 (phe)
7	Cyclo(-phe-pro)	$C_{14}H_{16}N_2O_2$	18.2	245.1288	217.1327	200.0634	172.1165	70.0654 (pro)	120.0799 (phe)
7’	Cyclo(-phe-pro)	$C_{14}H_{16}N_2O_2$	18.93	245.1288	217.1352	200.0962	172.1054	70.0657 (pro)	120.0806 (phe)
8	Cyclo(L-phe-L-ala)	$C_{12}H_{14}N_2O_2$	15.7	219.1133	*n.d.*	*n.d.*	146.0902	120.0798 (phe)	
9	Cyclo(L-val-L-ile)	$C_{11}H_{20}N_2O_2$	19.63	213.1599	*n.d.*	168.4196	140.1443	86.0979 ; 69.0657 (ile)	72.0821 (val)
10	Cyclo(L-val-L-leu)	$C_{11}H_{20}N_2O_2$	19.88	213.1599	*n.d.*	*n.d.*	140.1418	86.0966; 72.0454 (leu)	72.0804 (val)
11	Cyclo(L-leu-L-leu)	$C_{12}H_{22}N_2O_2$	22.9	227.1755	199.6368	182.1528	154.1552	86.0956 ; 72.0439 (leu)	
12	Cyclo(L-leu-L-ile)	$C_{12}H_{22}N_2O_2$	22.42	227.1755	*n.d.*	*n.d.*	154.1563	86.0965 ; 69.0703 (ile)	86.0965 ; 72.0421 (leu)
13	Cyclo(L-phe-L-phe)	$C_{18}H_{18}N_2O_2$	24.48	295.1443	267.1471	*n.d.*	222.1239	120.0805 (phe)	
14	Cyclo(L-ile-L-ala)	$C_9H_{16}N_2O_2$	14.02	185.1278	*n.d.*	*n.d.*	112.1096	86.0955 ; 69.0704 (ile)	
15	Cyclo(L-leu-L-ala)	$C_9H_{16}N_2O_2$	14.78	185.1278	*n.d.*	*n.d.*	112.1083	86.0974 ; 72.0436 (leu)	
16	Cyclo(L-phe-L-tyr)	$C_{18}H_{18}N_2O_3$	19.43	311.1399	*n.d.*	266.0963	238.1183	136.0721 (tyr)	120.0794 (phe)

n.d. not detected

Table S 2. Quantification of 2,5-diketopiperazines in 33 chocolate samples expressed as mg of diketopiperazine per kg of defatted chocolate powder.

	Cocoa Variety	Cyclo(L-pro-L-ile) 1	Cyclo(-pro-leu) 2	Cyclo(-pro-leu) 2'	Cyclo(L-pro-L-val) 3	Cyclo(L-phe-L-leu) 4	Cyclo(L-phe-L-ile) 5	Cyclo(L-phe-L-val) 6	Cyclo(-phe-pro) 7'	Cyclo(-phe-pro) 7	Cyclo(L-phe-L-ala) 8	Cyclo(L-ile-L-val) 9	Cyclo(L-leu-L-val) 10	Cyclo(L-leu-L-leu) 11	Cyclo(L-leu-L-ile) 12	Cyclo(L-phe-L-phe) 13	Cyclo(L-ile-L-ala) 14	Cyclo(L-leu-L-ala) 15	Cyclo(L-phe-L-tyr) 16	Sum
CL1	Unknown	83.3 ± 1.7	92.5 ± 3.1	55.5 ± 0.6	272.0 ± 5.7	12.7 ± 0.5	16.0 ± 0.7	10.4 ± 0.3	0.3 ± 0.0	13.2 ± 0.6	2.1 ± 0.1	58.8 ± 0.7	115.4 ± 2.7	109.0 ± 2.8	65.5 ± 0.9	11.7 ± 0.5	78.3 ± 1.7	76.4 ± 1.1	0.6 ± 0.1	1073.9
CL2	Unknown	81.9 ± 2.0	127.1 ± 3.7	62.2 ± 0.5	290.7 ± 12.1	13.7 ± 0.6	16.3 ± 0.8	10.8 ± 0.5	1.4 ± 0.1	26.7 ± 2.1	2.8 ± 0.2	58.0± 0.4	109.6 ± 2.7	100.4 ± 2.2	64.7 ± 0.5	13.6 ± 0.7	81.9 ± 1.4	82.4 ± 1.5	0.7 ± 0.1	1144.8
CL3	Unknown	71.4 ± 0.4	94.2 ± 0.4	59.1 ± 0.3	192.7 ± 1.3	7.9 ± 0.2	9.5 ± 0.2	5.8 ± 0.2	1.0 ± 0.0	9.3 ± 0.2	1.5 ± 0.0	55.2 ± 0.3	78.2 ± 0.4	74.4 ± 0.3	58.6 ± 0.5	8.5 ± 0.3	65.0 ± 0.4	65.3 ± 0.4	0.4 ± 0.1	858.0
SB1	Unknown	60.4 ± 0.6	70.4 ± 0.7	nd	106.1 ± 1.3	2.9 ± 0.1	3.9 ± 0.1	2.3 ± 0.1	nd	3.1 ± 0.1	0.5 ± 0.1	54.5 ± 0.4	68.4 ± 0.6	68.0 ± 0.4	56.2 ± 0.3	2.8 ± 0.1	58.4 ± 0.3	58.1 ± 0.5	nd	616.0
SB2	Unknown	59.4 ± 0.4	68.0 ± 0.4	nd	96.7 ± 0.3	2.4 ± 0.1	3.4 ± 0.1	2.0 ± 0.0	nd	3.2 ± 0.0	0.7 ± 0.1	54.7 ± 0.3	67.4 ± 0.3	66.2 ± 0.4	55.7 ± 0.2	2.5 ± 0.0	58.7 ± 0.4	58.6 ± 0.5	nd	599.7
SB3	Unknown	70.9 ± 0.8	91.3 ± 1.4	nd	160.8 ± 3.4	4.6 ± 0.4	6.7 ± 0.6	3.8 ± 0.3	nd	4.7 ± 0.3	0.5 ± 0.0	54.3 ± 0.5	82.6 ± 1.9	80.8 ± 1.2	57.2 ± 0.5	3.7 ± 0.4	57.8 ± 0.7	56.7 ± 0.8	nd	736.4
SB4	Criollo	61.3 ± 1.4	70.6 ± 1.9	nd	92.7 ± 3.2	3.1 ± 0.3	3.5 ± 0.4	1.7 ± 0.2	nd	2.1 ± 0.2	0.5 ± 0.0	54.8 ± 1.2	72.7 ± 2.3	70.1 ± 2.1	56.7 ± 1.4	2.9 ± 0.2	57.2 ± 1.2	57.2 ± 1	nd	607.1
SB5	Criollo	57.3 ± 1.5	60.5 ± 1.2	nd	70.1 ± 1.3	1.8 ± 0.1	1.9 ± 0.2	1.0 ± 0.1	nd	0.7 ± 0.0	0.2 ± 0.0	54.8 ± 1.3	63.9 ± 1.1	61.8 ± 1.2	56.2 ± 1.2	1.7 ± 0.1	56.4 ± 1.3	56.9 ± 1.2	nd	545.4
SB6	Criollo	52.7 ± 1.4	54.4 ± 1.4	nd	60.4 ± 1.5	1.0 ± 0.1	1.0 ± 0.1	0.6 ± 0.1	nd	0.7 ± 0.1	0.1 ± 0	0 ± 0	55 ± 1.3	53.9 ± 1.3	51.8 ± 1.4	1.1 ± 0.1	52.5 ± 1.3	52.8 ± 1.4	nd	438.0
SB7	Criollo	58.2 ± 0.5	62.6 ± 0.8	nd	75.9 ± 1.3	2.1 ± 0.2	2.3 ± 0.2	1.3 ± 0.1	nd	1.0 ± 0.1	0.2 ± 0.0	54.3 ± 0.2	65.6 ± 0.9	67.1 ± 1	55.9 ± 0.2	1.8 ± 0.1	56 ± 0.1	55.6 ± 0.2	nd	559.9
SB8	Criollo	60.3 ± 1.5	71.0 ± 1.9	nd	99.2 ± 4.6	3.7 ± 0.6	4.5 ± 0.8	2.7 ± 0.5	nd	2.4 ± 0.4	0.4 ± 0.1	54.2 ± 2.0	75.7 ± 2.7	71.1 ± 2.2	56.5 ± 1.8	2.8 ± 0.4	56.5 ± 1.8	57 ± 1.7	nd	617.8
SB9	Criollo	57.9 ± 1.5	61.9 ± 1.7	nd	74.4 ± 3.1	1.9 ± 0.1	2.2 ± 0.2	1.2 ± 0.1	nd	0.9 ± 0.1	0.2 ± 0.0	55.1 ± 1.3	65.5 ± 2.3	64.0 ± 2.1	56.1 ± 1.3	1.7 ± 0.2	56.4 ± 1.4	56.2 ± 1.5	nd	555.6
SB10	Criollo	68.5 ± 1.1	88.4 ± 2.4	nd	136.4 ± 4.7	5.3 ± 0.5	6.0 ± 0.4	3.3 ± 0.2	nd	4.0 ± 0.3	0.6 ± 0.1	55.8 ± 0.4	83.9 ± 2.2	78.7 ± 2	59.2 ± 0.6	4.3 ± 0.3	59.2 ± 0.4	58.9 ± 0.4	nd	712.4
SB11	Trinitario	60.6 ± 1.8	68.2 ± 3.1	nd	98.8 ± 8.8	3.2 ± 0.9	3.3 ± 0.9	2.0 ± 0.5	nd	2.3 ± 0.4	0.5 ± 0.1	53.8 ± 1.0	68.8 ± 4.3	69.4 ± 4.2	56.3 ± 1.3	2.6 ± 0.6	56.8 ± 1.4	56.1 ± 1.2	0.2 ± 0.1	602.8
SB12	Trinitario	70.4 ± 3.5	90.1 ± 6.4	nd	163.2 ± 16.8	6.9 ± 1.1	8.2 ± 1.1	5.9 ± 0.9	nd	5.9 ± 0.9	1.1 ± 0.2	58.0 ± 1.5	93.9 ± 6.7	82.3 ± 5	62.1 ± 1.8	4.8 ± 0.7	64.1 ± 2.1	66.7 ± 2.8	0.2 ± 0.0	783.9
SB13	Trinitario	77.7 ± 1	106.7 ± 2.9	nd	193.7 ± 3.3	11.0 ± 0.5	14.5 ± 0.8	8.7 ± 0.4	nd	8.5 ± 0.3	1.3 ± 0.1	58.9 ± 0.5	117.0 ± 3.5	104.6 ± 3.1	65.0 ± 1.0	5.8 ± 4.6	68.0 ± 1	69.3 ± 1.3	0.5 ± 0.0	911.3
SB14	Trinitario	59.1 ± 1.2	65.7 ± 1.6	nd	81.0 ± 3.2	3.1 ± 0.2	3.3 ± 0.3	2.0 ± 0.2	nd	1.6 ± 0.1	0.4 ± 0.0	54.6 ± 0.9	70.4 ± 2	69 ± 2	56.7 ± 1	2.5 ± 0.2	57.7 ± 1.3	56.9 ± 1.1	0.2 ± 0.1	584.3
SB15	Trinitario	70.4 ± 0.9	93.0 ± 1.5	nd	161.6 ± 4.2	7.4 ± 0.3	10.8 ± 0.6	7.1 ± 0.3	nd	5.2 ± 0.3	1.2 ± 0.1	60.6 ± 1.0	114.5 ± 3.1	102.9 ± 1.7	64.7 ± 1.2	4.9 ± 0.3	66.1 ± 1.2	68.0 ± 1.2	0.3 ± 0.0	838.9
SB16	Criollo/Trinitario	71.5 ± 1.2	93.2 ± 2.5	nd	190.0 ± 5.3	16.9 ± 0.4	19.4 ± 0.3	13.4 ± 0.3	nd	12.3 ± 0.4	3.4 ± 0.1	58.3 ± 0.4	109.2 ± 1.9	94.0 ± 1.0	65.1 ± 0.2	15.3 ± 0.3	80.4 ± 1.1	81.2 ± 0.9	1.3 ± 0.0	924.9
SB17	Trinitario	56.9 ± 3.3	62.9 ± 3.1	nd	78.8 ± 3.3	3.4 ± 0.1	4.2 ± 0.1	2.2 ± 0.1	nd	1.5 ± 0	0.3 ± 0.0	52.1 ± 3.3	69.9 ± 3.1	71.3 ± 3	54.4 ± 3.1	2.9 ± 0.1	54.9 ± 3.2	53.7 ± 3.2	0.2 ± 0	569.7
SB18	Trinitario	70.8 ± 1.4	83.7 ± 3.6	nd	125.5 ± 10.8	3.7 ± 0.7	5.2 ± 0.9	3.3 ± 0.6	nd	4.4 ± 1	0.5 ± 0.1	52.5 ± 0.2	72.1 ± 3	69.8 ± 2.7	54.4 ± 0.2	2.8 ± 0.6	56.1 ± 0.9	55.8 ± 0.8	0.1 ± 0	660.7
SB19	Trinitario	86.0 ± 3.5	124 ± 5.6	nd	271.9 ± 16.6	9.3 ± 0.5	14.6 ± 0.6	8.3 ± 0.4	nd	9.8 ± 0.6	1.3 ± 0.1	60.5 ± 2.3	123.8 ± 4.6	110.4 ± 4.3	63.7 ± 2.4	7.2 ± 0.4	68.9 ± 2.9	70.3 ± 2.9	0.4 ± 0.0	1030.4
SB20	Trinitario	77.6 ± 1.9	105.1 ± 3.3	nd	173 ± 5.5	8.1 ± 0.5	10.8 ± 0.3	6.4 ± 0.3	nd	6.1 ± 0.3	0.9 ± 0.0	56.9 ± 3.5	111 ± 8.0	101.4 ± 7.1	61.6 ± 4.1	6.1 ± 0.3	60.3 ± 4.2	59.7 ± 4.3	0.4 ± 0.1	845.4
SB21	Trinitario	80.5 ± 5.4	113.3 ± 15.7	nd	207 ± 26.5	7.2 ± 1.2	9.8 ± 1.8	5.6 ± 1.0	nd	8.5 ± 1.5	0.9 ± 0.1	56.4 ± 1.6	105.9 ± 10	96.7 ± 8.2	59.8 ± 2.2	5.9 ± 1.0	62.2 ± 2.6	61.5 ± 2.6	0.3 ± 0.0	881.4
SB22	Forastero	81.3 ± 0.3	113.0 ± 1.2	nd	230.4 ± 3.3	12.5 ± 0.3	17.3 ± 0.2	10.3 ± 0.2	nd	12.4 ± 0.2	2.1 ± 0.1	59.7 ± 0.1	117.6 ± 1.0	102.0 ± 0.7	65.4 ± 0.2	11.1 ± 0.2	74.2 ± 0.2	74.1 ± 0.6	0.8 ± 0.1	984.1
SB23	Forastero	85.9 ± 1.3	117.8 ± 2.7	nd	227.7 ± 7.4	12.8 ± 0.7	15.1 ± 0.7	8.5 ± 0.6	nd	9.5 ± 0.6	1.7 ± 0.1	58.7 ± 0.5	128.8 ± 3.2	133.2 ± 2.2	68.0 ± 0.2	11.2 ± 0.5	70.5 ± 0.3	66.2 ± 0.7	0.7 ± 0.1	1016.3
SB24	Forastero	73.6 ± 3.4	99.3 ± 5.7	nd	198.9 ± 11.9	7.6 ± 1	10.6 ± 1.4	6.5 ± 0.6	nd	9.7 ± 0.9	1.4 ± 0.1	57.1 ± 0.8	93.5 ± 4.8	89.1 ± 5.7	61.2 ± 1.4	6.8 ± 0.8	66.7 ± 1.7	66.4 ± 1.0	0.3 ± 0.1	848.7
SB25	Forastero	80.3 ± 0.5	109.0 ± 0.9	nd	234.9 ± 4	11.0 ± 0.2	12.6 ± 0.2	8.7 ± 0.1	nd	8.6 ± 0.2	1.8 ± 0.0	57.8 ± 0.4	107.9 ± 0.1	96.7 ± 0.4	65.5 ± 0.3	8.5 ± 0.0	70.1 ± 0.5	69.4 ± 0.6	0.5 ± 0.1	943.4
SB26	Unknown	94.1 ± 1.2	137.0 ± 2.0	nd	337.7 ± 8	9.4 ± 0.7	13.6 ± 0.3	8.7 ± 0.3	nd	11.7 ± 0.5	1.9 ± 0.1	59.9 ± 0.2	109.1 ± 1.5	104.8 ± 1.5	65.7 ± 0.2	7.6 ± 0.5	71.0 ± 0.9	73.8 ± 0.6	0.4 ± 0.0	1106.3
Ref1	Forastero	78.2 ± 3.1	110.3 ± 3.9	nd	228.3 ± 7.8	5.5 ± 0.2	8.4 ± 0.4	4.9 ± 0.2	nd	7.2 ± 0.3	0.7 ± 0.0	53.9 ± 2.6	89.6 ± 3.5	89.1 ± 3.1	57.3 ± 2.7	4.6 ± 0.2	59.1 ± 2.6	57.7 ± 2.5	nd	854.8
Ref2	Criollo	57.8 ± 0.2	63.7 ± 0.6	nd	74.5 ± 0.4	1.9 ± 0.1	2.0 ± 0.1	1.1 ± 0.1	nd	1.2 ± 0	0.2 ± 0.0	65.0 ± 0.1	65.3 ± 0.1	55.5 ± 0.1	60.9 ± 0.1	0.4 ± 0.1	55.4 ± 0.2	53.4 ± 0.3	nd	519.2
Ref3	Trinitario	63.6 ± 1.2	72.4 ± 0.4	nd	93.2 ± 2.8	3.6 ± 0.5	5.1 ± 0.7	2.7 ± 0.4	nd	2.1 ± 0.3	0.3 ± 0.1	54.8 ± 1.9	75.6 ± 6.1	72.6 ± 5.7	57.1 ± 2.5	2.8 ± 0.4	56.4 ± 2.3	55.7 ± 2	nd	617.9
Ref4	Trinitatio	64.3 ± 2.2	79.1 ± 2.4	nd	111.6 ± 3.3	4.1 ± 0.1	5.9 ± 0.1	2.7 ± 0.1	nd	3.5 ± 0.1	0.4 ± 0.1	54.0 ± 2.3	79.1 ± 2.5	80.4 ± 2.6	56 ± 2.4	3.7 ± 0.1	55.3 ± 2.1	54.8 ± 1.9	0.2 ± 0	655.2

nd: not detected

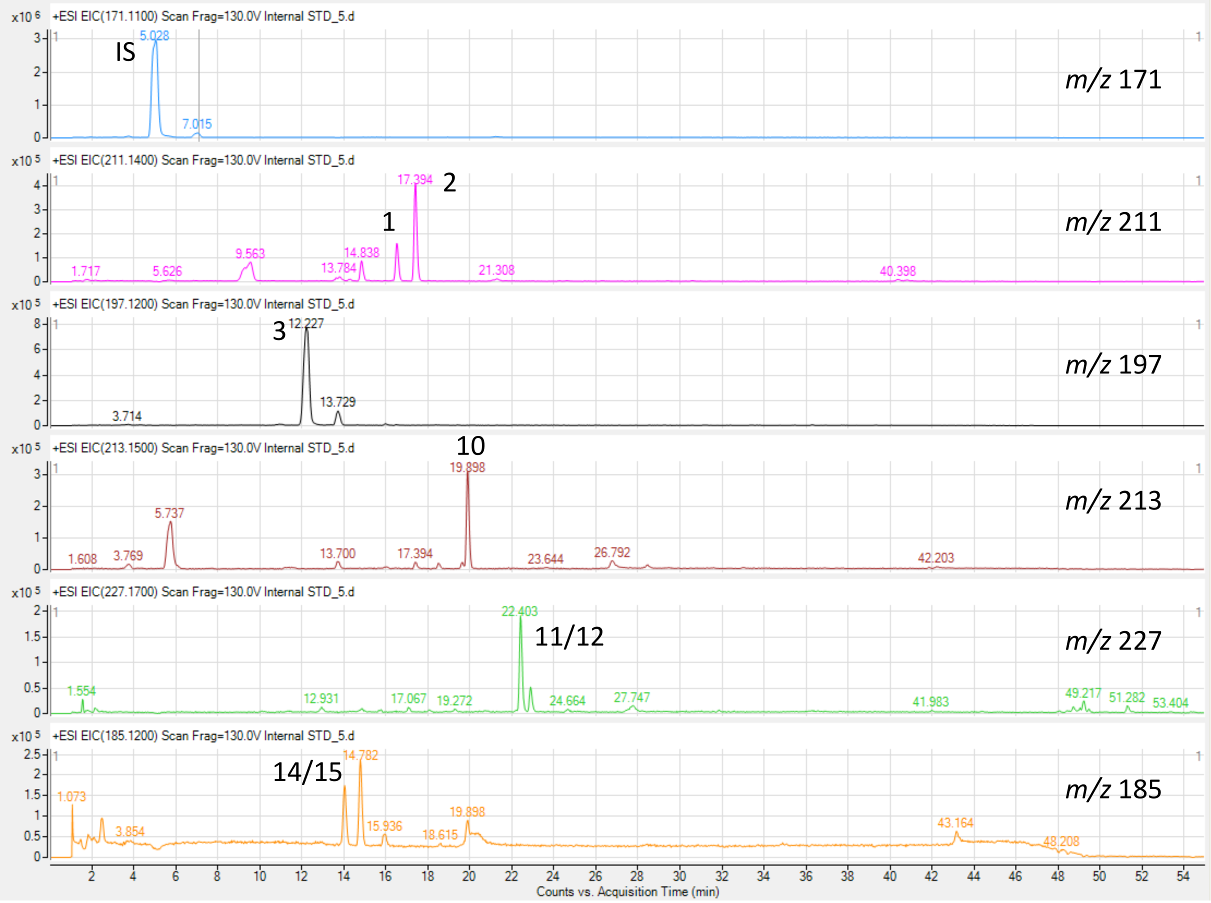

Figure S 1. Quantification of selected diketopiperazines using trans-cyclo(D-ala-L-val) as internal standard (IS)

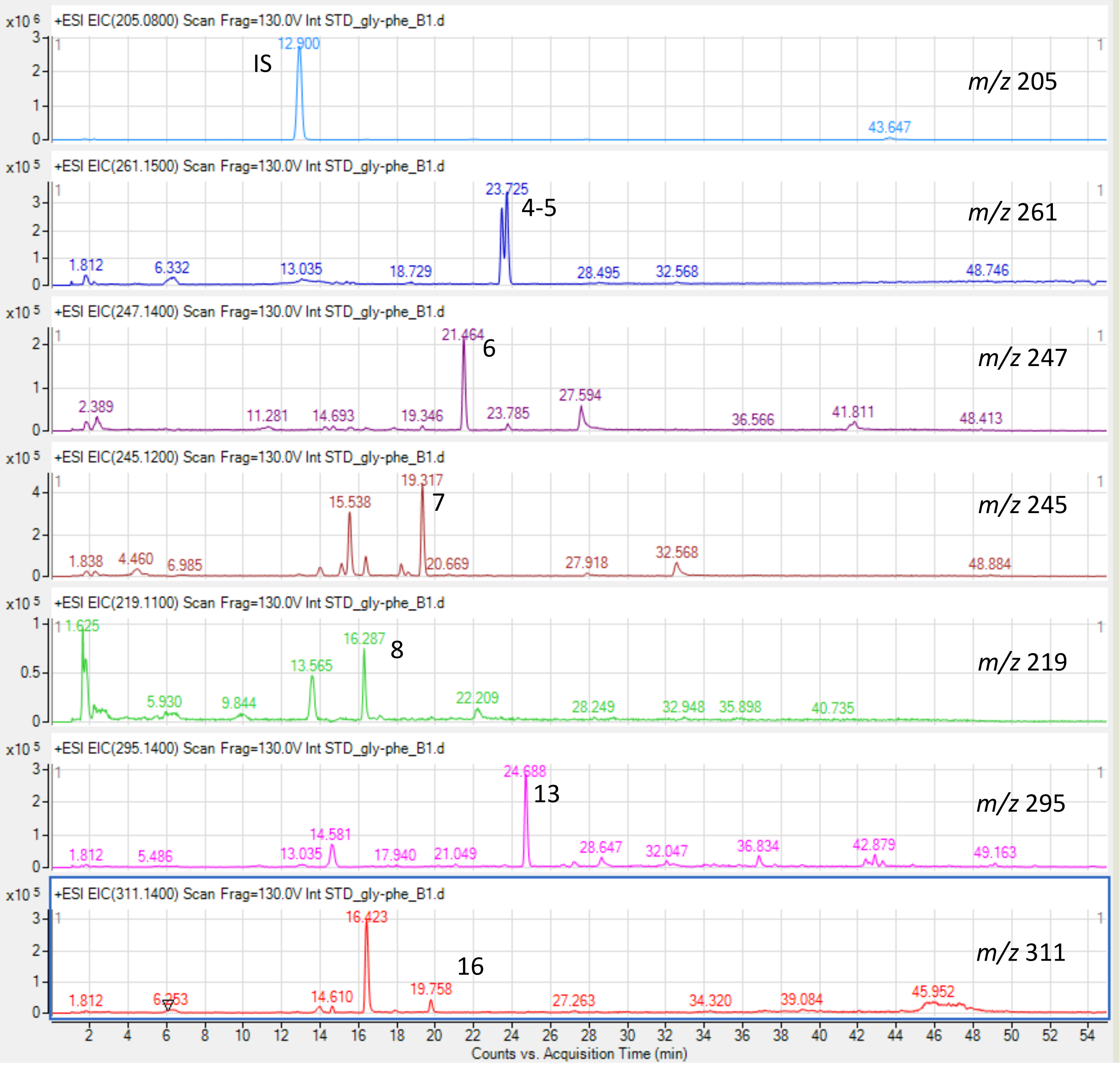

Figure S 2. Quantification of selected diketopiperazines using cyclo(-gly-phe) as internal standard (IS).

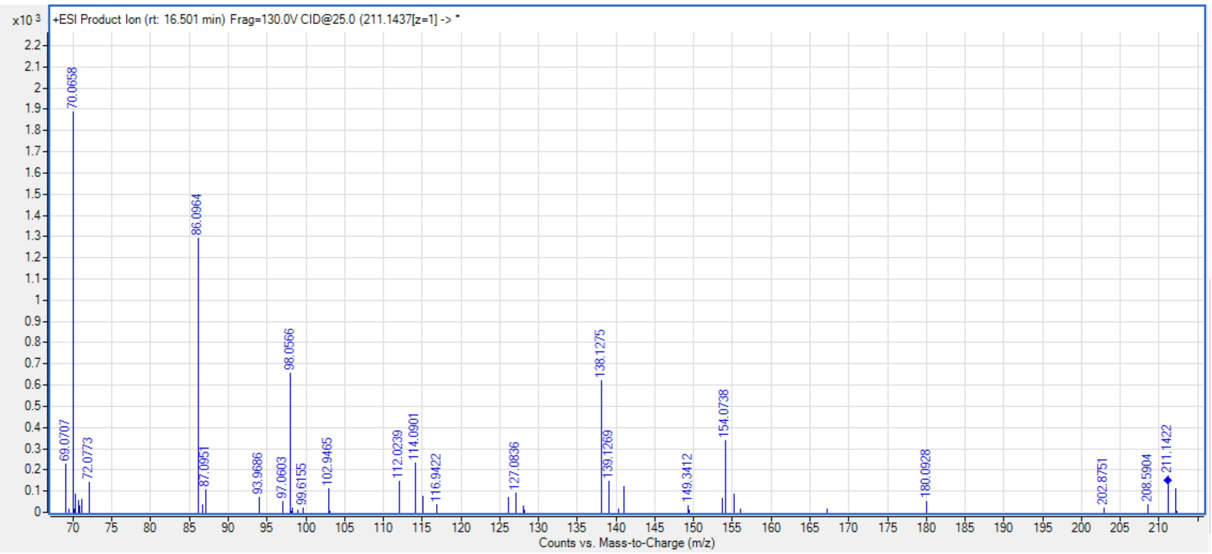

Figure S 3. MS/MS spectra of cyclo(L-pro-L-ile) ***1***

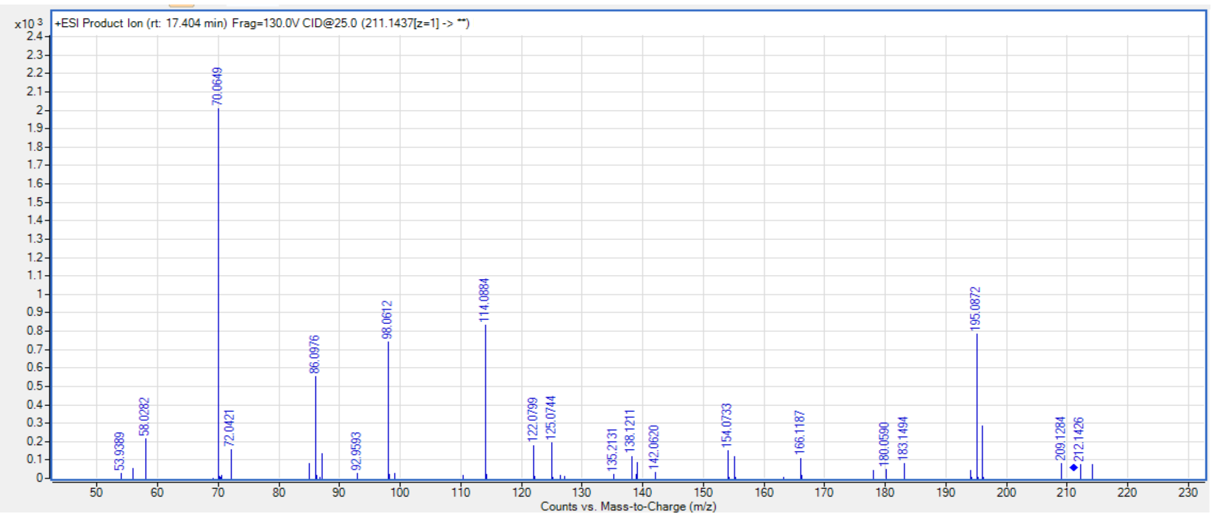

Figure S 4. MS/MS spectra of cyclo(-pro-leu) ***2***

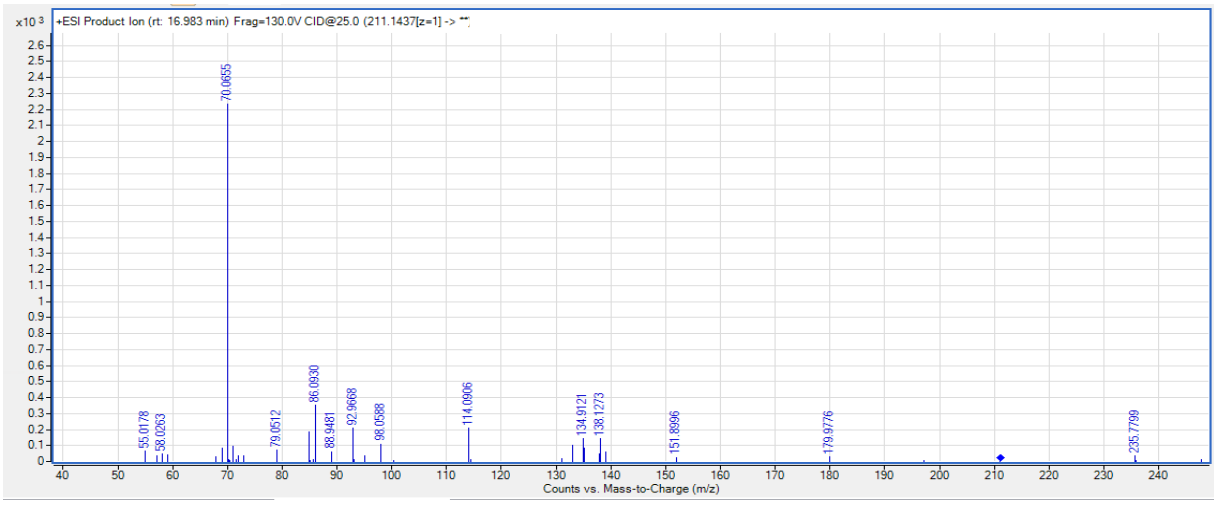

Figure S 5. MS/MS spectra of cyclo(-pro-leu) ***2'***

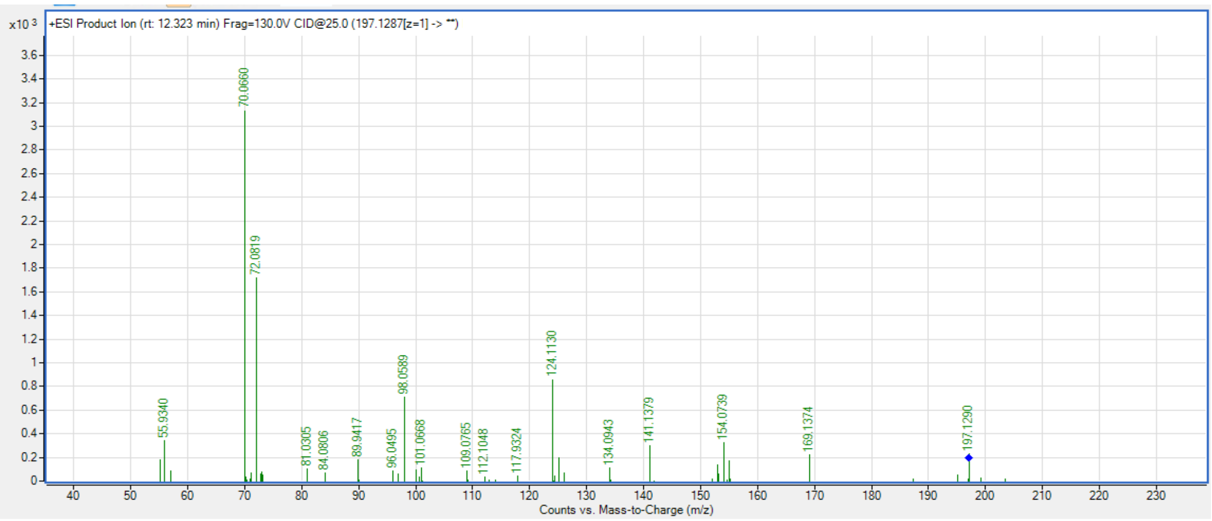

Figure S 6. MS/MS spectra of cyclo(L-pro-L-val) ***3***

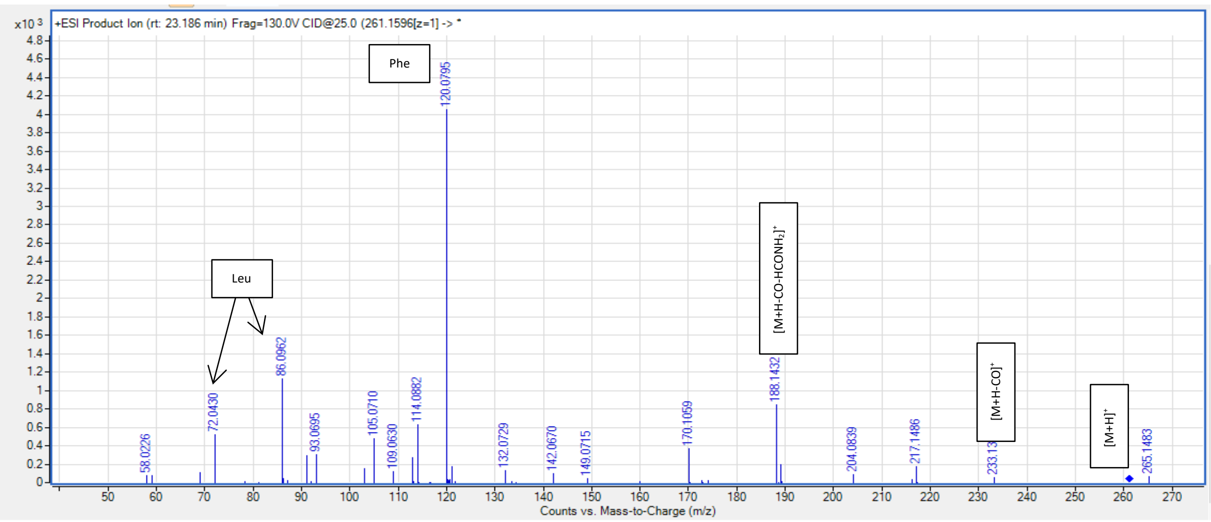

Figure S 7. MS/MS spectra of cyclo(L-phe-L-leu) ***4***

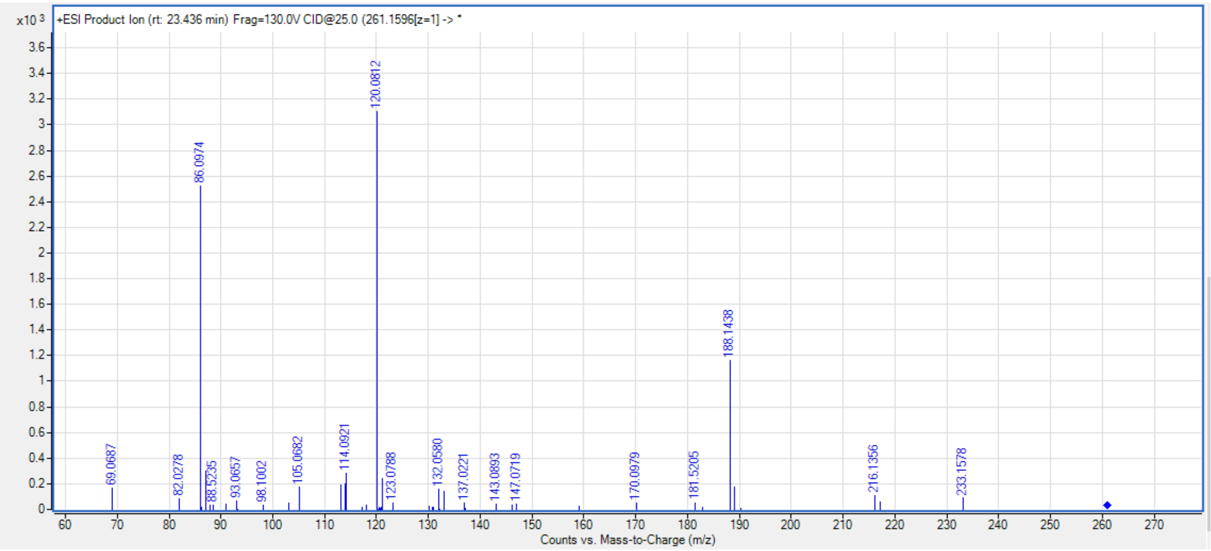

Figure S 8. MS/MS spectra of cyclo(L-phe-L-ile) ***5***

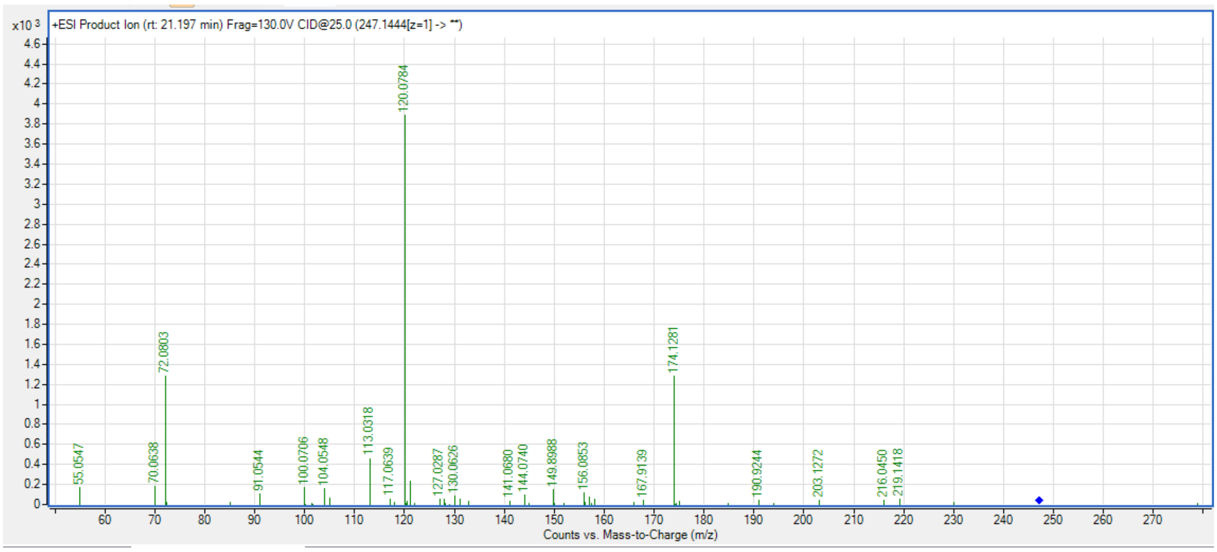

Figure S 9. MS/MS spectra of cyclo(L-phe-L-val) ***6***

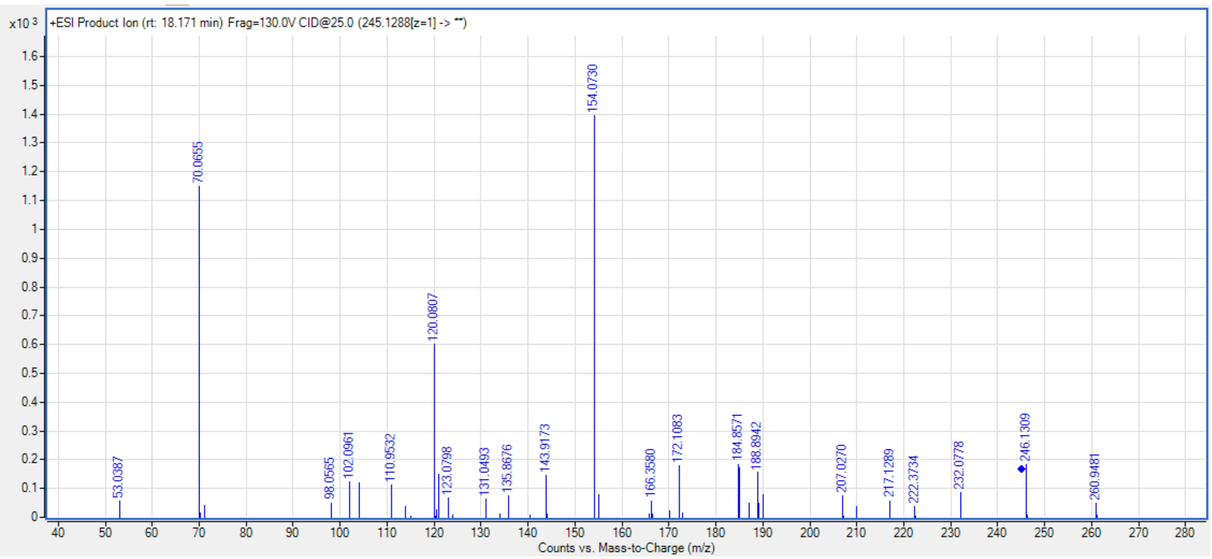

Figure S 10. MS/MS spectra of cyclo(-phe-pro) ***7***

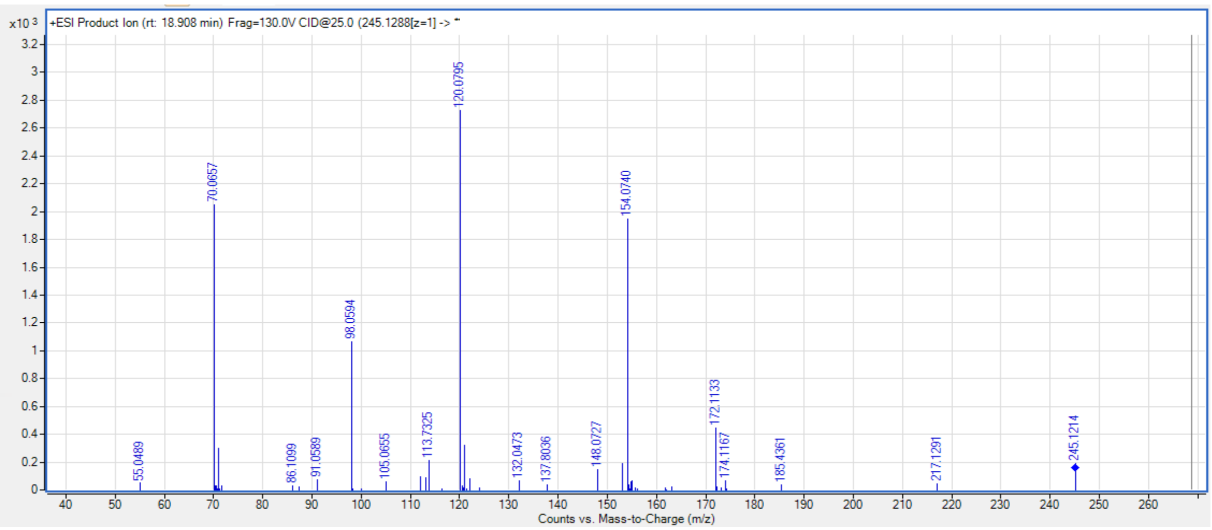

Figure S 11. MS/MS spectra of cyclo(-phe-pro) ***7’***

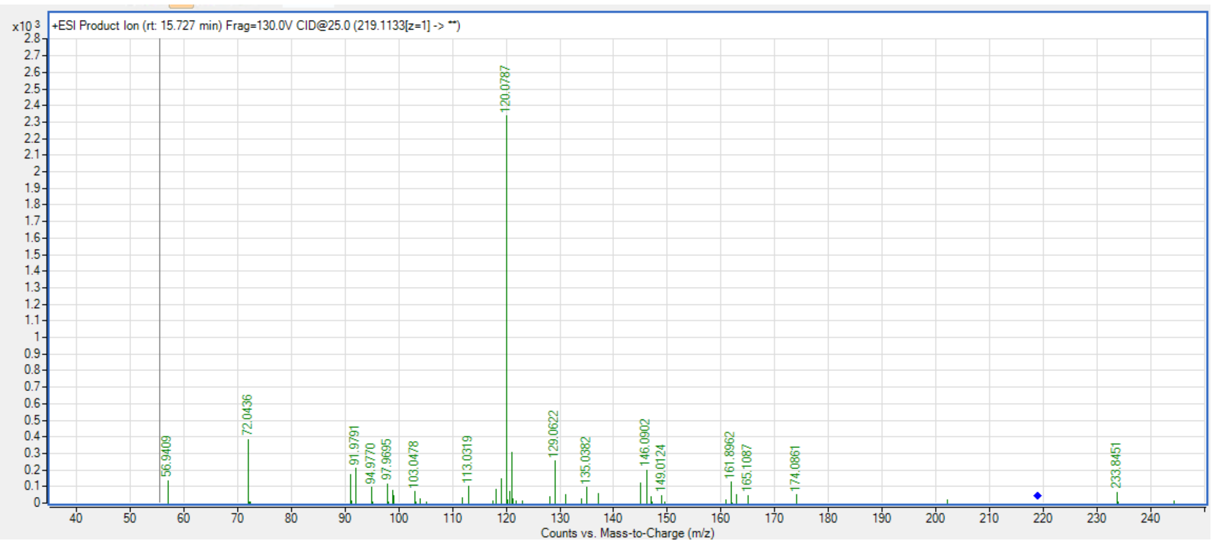

Figure S 12. MS/MS spectra of cyclo(L-phe-L-ala) ***8***

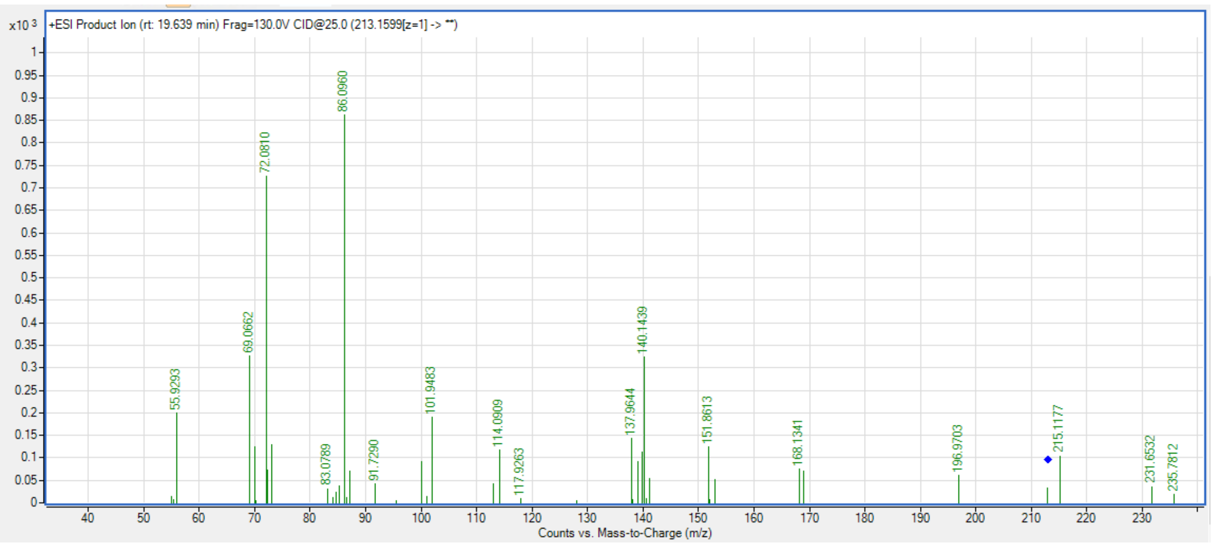

Figure S 13. MS/MS spectra of cyclo(L-val-L-ile) ***9***

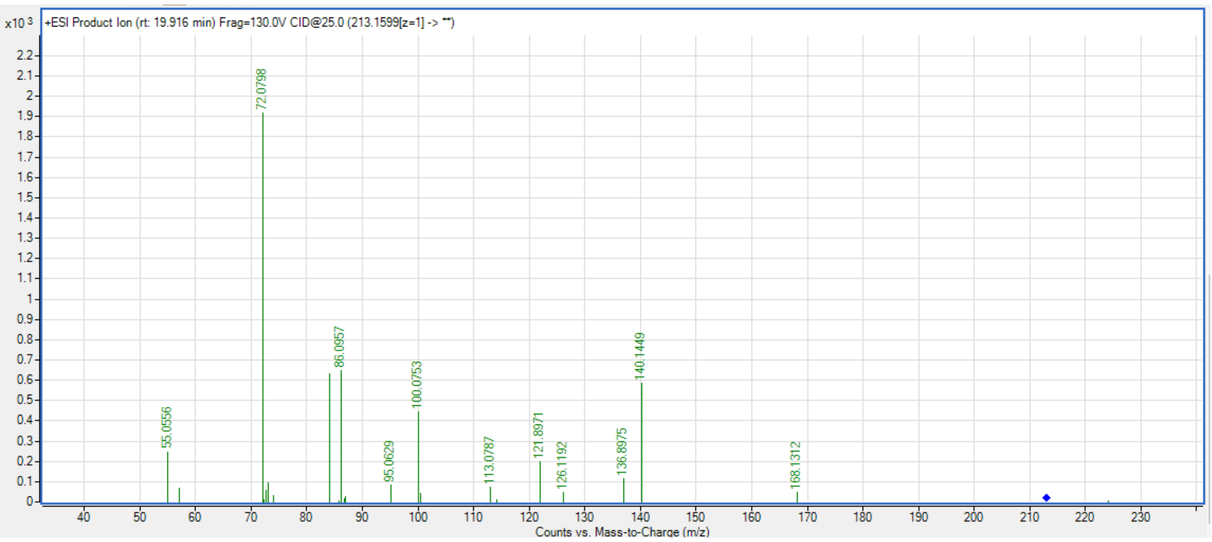

Figure S 14. MS/MS spectra of cyclo(L-val-L-leu) ***10***

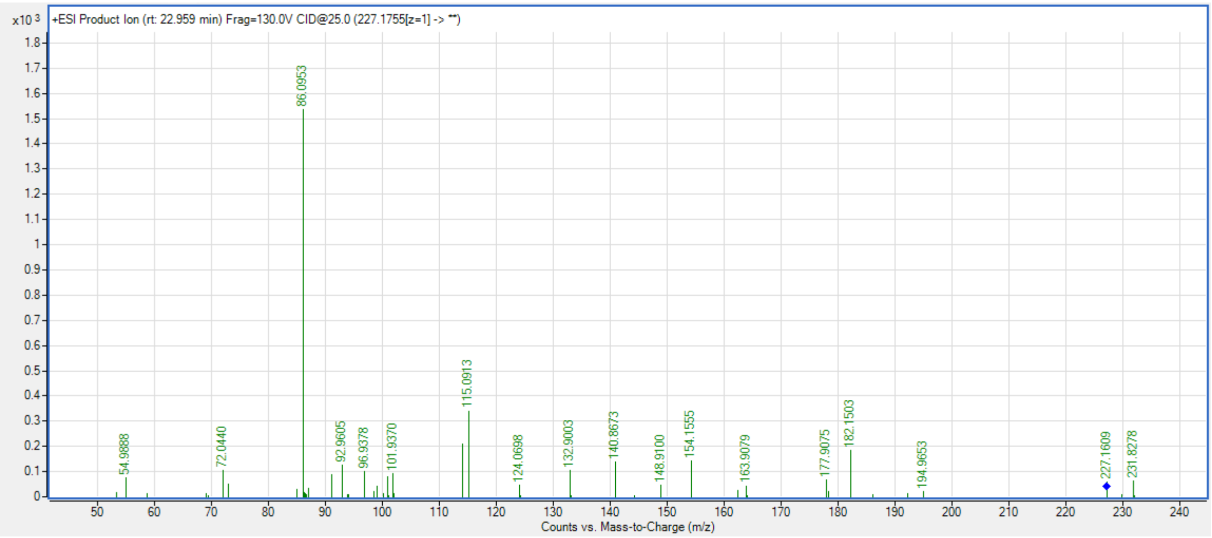

Figure S 15. MS/MS spectra of cyclo(L-leu-L-leu) ***11***

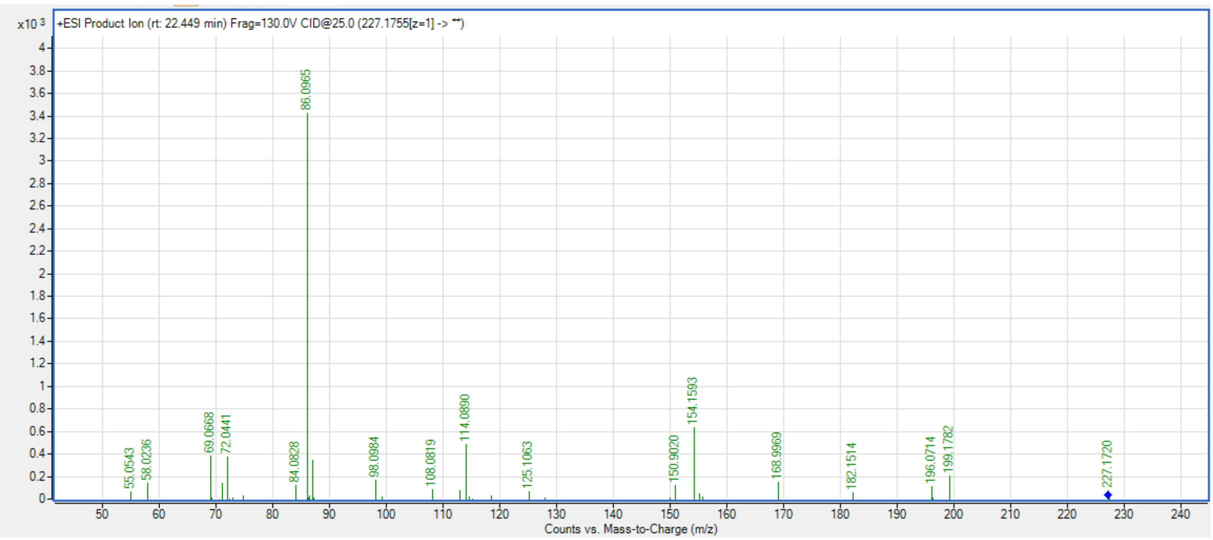

Figure S 16. MS/MS spectra of cyclo(L-leu-L-ile) ***12***

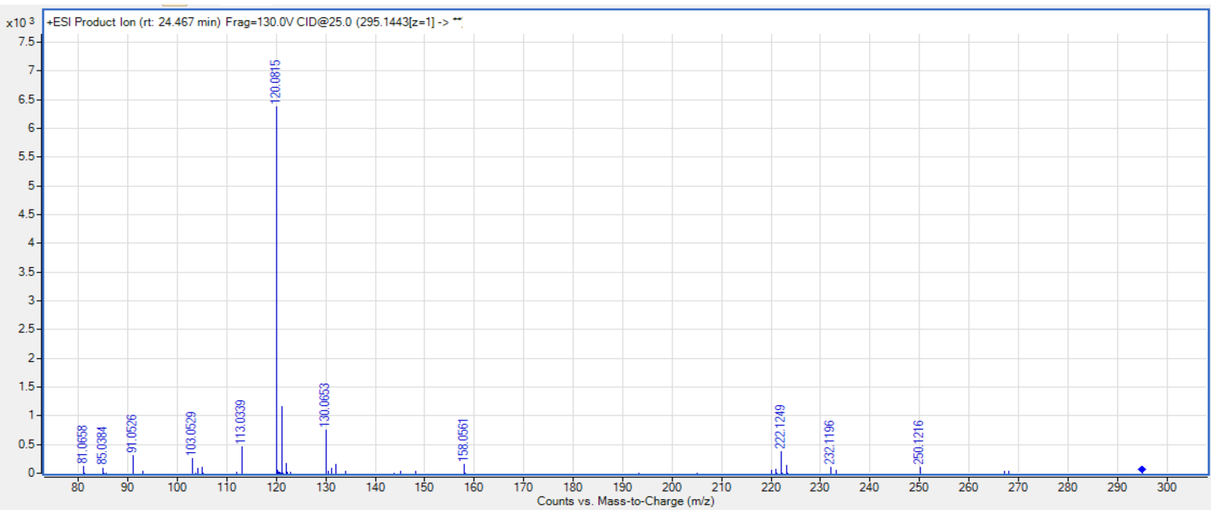

Figure S 17. MS/MS spectra of cyclo(L-phe-L-phe) ***13***

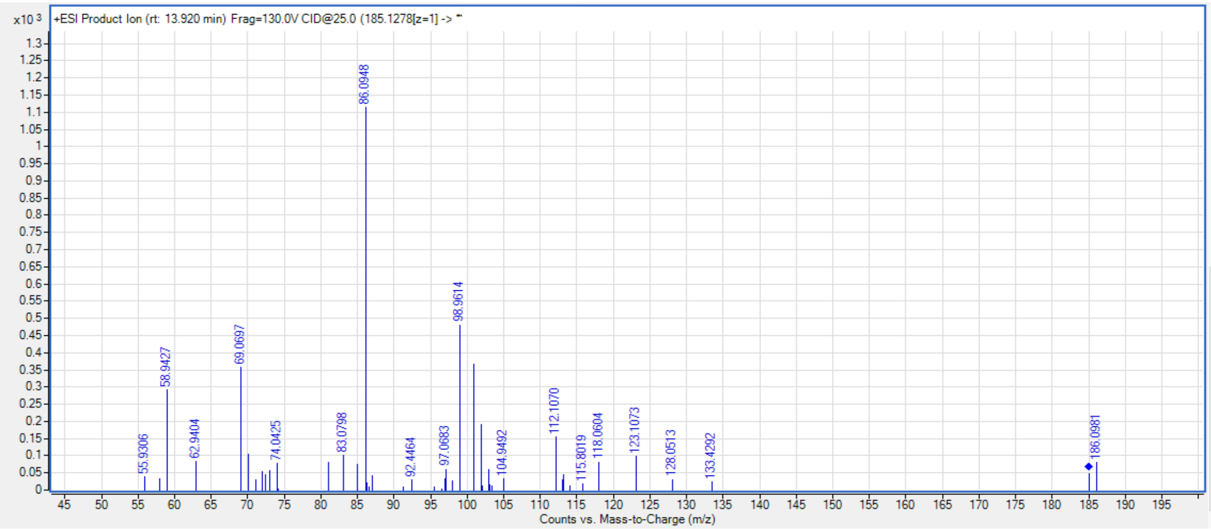

Figure S 18. MS/MS spectra of cyclo(L-ile-L-ala) ***14***

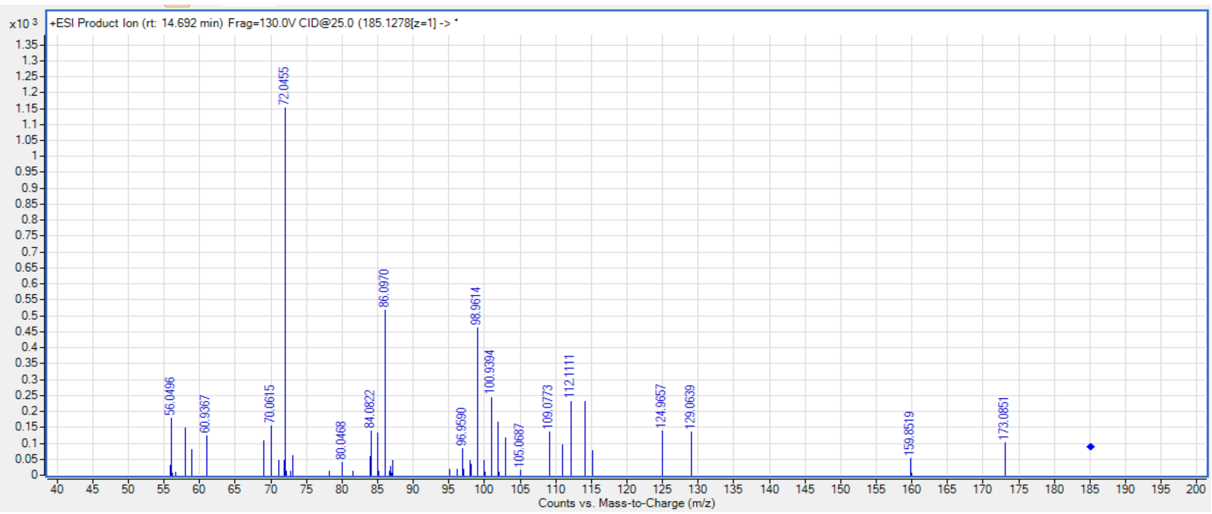

Figure S 19. MS/MS spectra of cyclo(L-leu-L-ala) ***15***

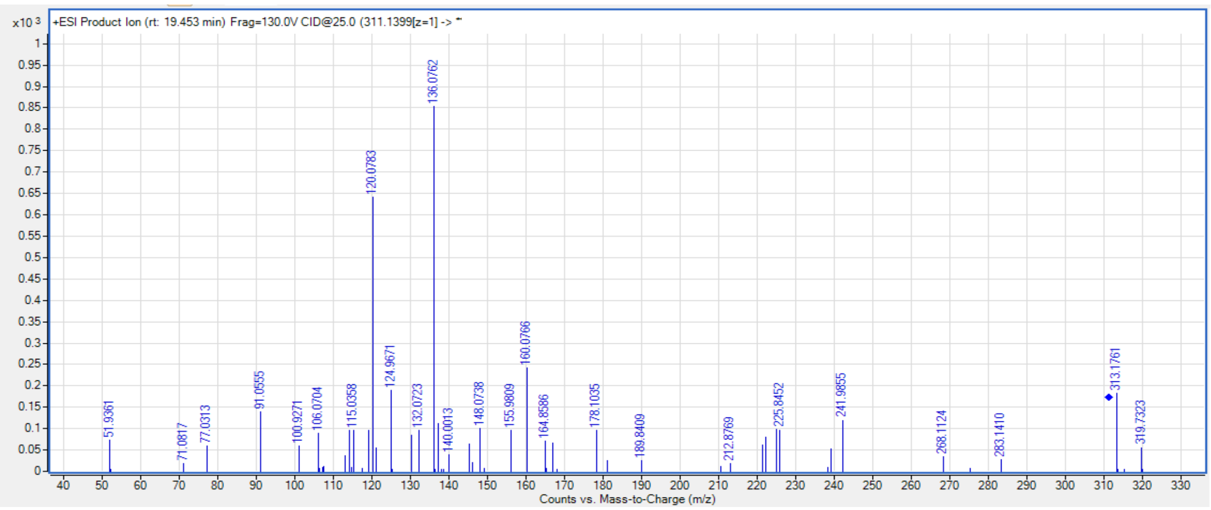

Figure S 20. MS/MS spectra of cyclo(L-phe-L-tyr) ***16***
